# Antimalarial drug discovery against malaria parasites through haplopine modification: An advanced computational approach

**DOI:** 10.1111/jcmm.17940

**Published:** 2023-09-19

**Authors:** Shopnil Akash, Guendouzi Abdelkrim, Imren Bayil, Md. Eram Hosen, Nobendu Mukerjee, Abdullah F. Shater, Fayez M. Saleh, Ghadeer M. Albadrani, Muath Q. Al‐Ghadi, Mohamed M. Abdel‐Daim, Tuğba Taşkin Tok

**Affiliations:** ^1^ Department of Pharmacy Faculty of Allied Health Sciences, Daffodil International, University Dhaka Bangladesh; ^2^ Laboratory of Chemistry, Synthesis, Properties and Applications. (LCSPA) University of Saida Saïda Algeria; ^3^ Department of Bioinformatics and computational biology Gaziantep University Gaziantep Turkey; ^4^ Professor Joarder DNA and Chromosome Research Laboratory, Department of Genetic Engineering and Biotechnology University of Rajshahi Rajshahi Bangladesh; ^5^ Department of Microbiology West Bengal State University Kolkata India; ^6^ Department of Health Sciences Novel Global Community Educational Foundation Hebersham Australia; ^7^ Department of Medical Laboratory Technology, Faculty of Applied Medical Sciences University of Tabuk Tabuk Saudi Arabia; ^8^ Department of Medical Microbiology, Faculty of Medicine University of Tabuk Tabuk Saudi Arabia; ^9^ Department of Biology, College of Science Princess Nourah bint Abdulrahman University Riyadh Saudi Arabia; ^10^ Department of Zoology, College of Science King Saud University Riyadh Saudi Arabia; ^11^ Department of Pharmaceutical Sciences, Pharmacy Program Batterjee Medical College Jeddah Saudi Arabia; ^12^ Pharmacology Department, Faculty of Veterinary Medicine Suez Canal University Ismailia Egypt

**Keywords:** ADMET, Apicoplast DNA polymerase, DFT, malaria, molecular docking, *pathogenesis*

## Abstract

The widespread emergence of antimalarial drug resistance has created a major threat to public health. Malaria is a life‐threatening infectious disease caused by *Plasmodium* spp., which includes Apicoplast DNA polymerase and *Plasmodium falciparum* cysteine protease falcipain‐2. These components play a critical role in their life cycle and metabolic pathway, and are involved in the breakdown of erythrocyte hemoglobin in the host, making them promising targets for anti‐malarial drug design. Our current study has been designed to explore the potential inhibitors from haplopine derivatives against these two targets using an in silico approach. A total of nine haplopine derivatives were used to perform molecular docking, and the results revealed that Ligands 03 and 05 showed strong binding affinity compared to the control compound atovaquone. Furthermore, these ligand‐protein complexes underwent molecular dynamics simulations, and the results demonstrated that the complexes maintained strong stability in terms of RMSD (root mean square deviation), RMSF (root mean square fluctuation), and Rg (radius of gyration) over a 100 ns simulation period. Additionally, PCA (principal component analysis) analysis and the dynamic cross‐correlation matrix showed positive outcomes for the protein‐ligand complexes. Moreover, the compounds exhibited no violations of the Lipinski rule, and ADMET (absorption, distribution, metabolism, excretion, and toxicity) predictions yielded positive results without indicating any toxicity. Finally, density functional theory (DFT) and molecular electrostatic potential calculations were conducted, revealing that the mentioned derivatives exhibited better stability and outstanding performance. Overall, this computational approach suggests that these haplopine derivatives could serve as a potential source for developing new, effective antimalarial drugs to combat malaria. However, further in vitro or in vivo studies might be conducted to determine their actual effectiveness.

## 1 INTRODUCTION

Malaria is a lethal disease that can occasionally be deadly, and it is transmitted by a parasite that typically infects certain types of mosquitos that bites on people. Currently, about half of the world's population is at risk of life‐threatening infectious malaria disease caused by protozoa of the genus Plasmodium that spread by the bite of the female mosquito named Anopheles.^1^, ^2^ There are five species of Plasmodium namely *P. vivax, P. malariae P. falciparum, P. ovale wallikeri* and *P. ovale curtisi* are responsible for malaria infection in humans whereas *P. falciparum* are the most common and lethal.^3^ Malaria may develop symptoms like fever, headache, chills, vomiting, nausea, diarrhoea, muscle aches and tiredness but not specific, sometimes it causes anaemia, jaundice and organ failures eventually leading to coma and death.^3^, ^4^ In 2022, the world malaria report showed an increase cases of 247 million malarias with 619,000 death occurred in 2021 compared to the 568,000 death with 245 million cases in 2020.^5^

The artemether‐lumefantrine (Coartem®), atovaquone‐proguanil (Malarone™), quinine, mefloquine, artemisinin, chloroquine and primaquine are the preferred antimalarial drugs.^6^, ^7^ However, due to indiscriminate use of these drugs and other factors antimalarial drug resistance has been increased that resulted recurrent parasitemia, a cumulative risk of anaemia and the development of a serious and lethal disease.^8^

In the last 15 years, there has been a significant improvement in the treatment of malaria and use vaccine to overcome this problem. The only registered malaria vaccine is RTS, S (Mosquirix®, GlaxoSmithKline) that confers only uncertain, short‐term protection^9^, ^10^ whereas BCG vaccination is reported with protection against malaria.^11^ Research on malaria vaccines has, to yet, mostly concentrated on the production of potent antibody or T‐cell responses. However, new research indicates that some vaccines, such as the BCG vaccine may cause long‐lasting alterations in the innate immune system that have non‐specific memory properties, additionally BCG‐induced ‘trained immunity’ which is also alterations in innate immune cells.^12^, ^13^

It is now obvious that new and improved anti‐malaria drug discovery are needed for controlled. Most of the *Plasmodium* spp. contains unusual organelle called the apicoplast that evolutionarily related to the chloroplast and involves in a number of metabolic processes, including biosynthesis of fatty acids, iron–sulfur clusters, haem and isoprenoids.^14^, ^15^ An enzyme apicoplast DNA polymerase replicates and repairs the genome of apicoplast and as this parasite relies on apicoplast, so, any defects in apicoplast metabolism or its inability to multiply and divide, cause Plasmodium to die in the blood and liver stages of infection.^16^ Therefore, Apicoplast DNA polymerase is considered a promising drug target for developing anti‐malaria drug. Another promising drug target to develop anti‐malaria chemotherapy is the *Plasmodium falciparum* cysteine protease falcipain‐2, is one of four papain‐family cysteine proteases known as falcipains‐2 expressed by malaria parasite *Plasmodium falciparum*.^17^ At the erythrocyte stage of the parasite the falcipains‐2 is over‐expressed and involved in the degradation of host erythrocyte haemoglobin and erythrocyte membrane skeletal proteins.^18^

Haplopine is a natural alkaloids and biologically active compounds mostly found in *Haplophyllum bucharicum*, *Haplophyllum cappadocicum*, *D. dasycarpus* and other organism, effectively possess antioxidant, anti‐inflammatory, photo‐activated antimicrobial activities and effective against potent melanogenesis and other skin diseases without any toxicity.^19^, ^20^, ^21^

Herein, our aims to develop a new effective compound from haplopine derivatives to combat malaria by targeting Apicoplast DNA polymerase and *Plasmodium falciparum* cysteine protease falcipain‐2 through density functional theory (DFT), molecular docking, molecular dynamics (MD), principal component analysis (PCA) analysis and absorption, distribution, metabolism, elimination and toxicity (ADMET) prediction.

## 2 METHODOLOGY

### 2.1 Preparation of ligand

**FIGURE 1:**
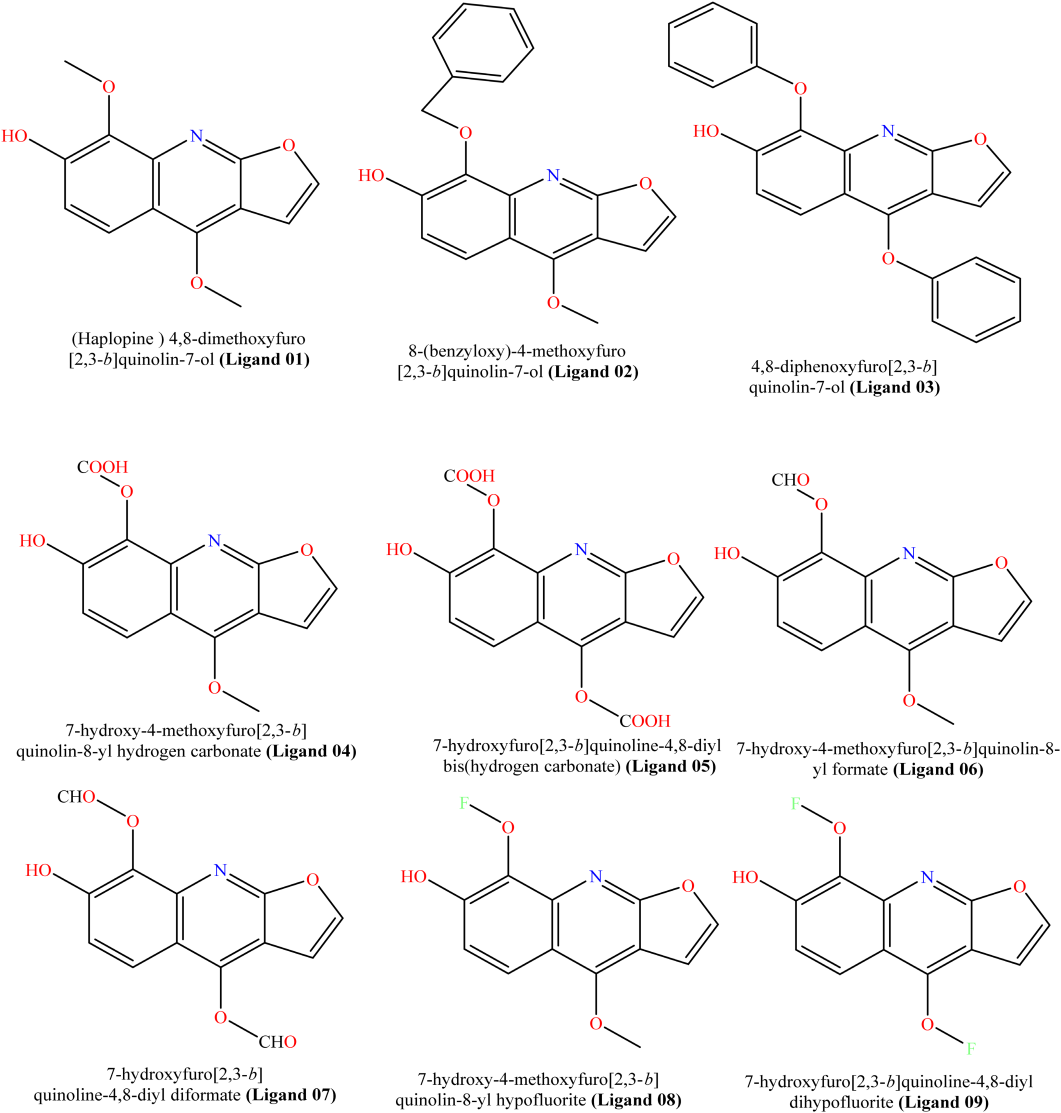
Molecular structures of haplopine derivatives.

Haplopine is an alkaloid and initially it is taken as parent compounds after that it has been modified and designed with Chem Bio Draw 12.0.02(Figure 1). Then, they have been optimized by using material studio application in DFT method of DMol code.^22^ When, optimization is done, the haplopine derivatives were saved as PDB files types for further investigation such as molecular docking, ADMET and related computational work.

### 2.2 Protein preparation and molecular docking study

**FIGURE 2:**
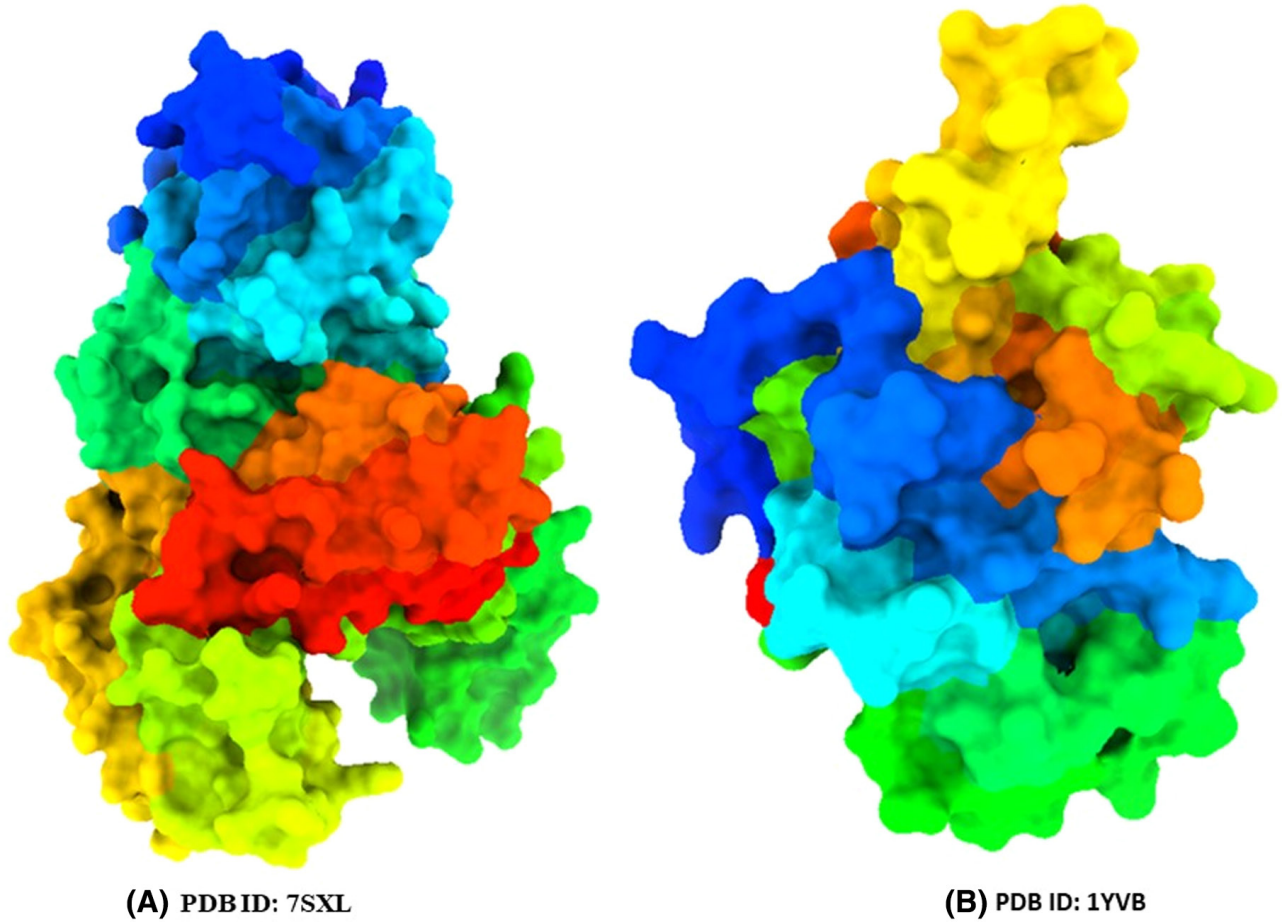
Three‐dimensional protein structure. Apicoplast DNA polymerase (A), and *Plasmodium falciparum* cysteine protease falcipain‐2 (B).

The targeted Malarial proteins (Apicoplast DNA polymerase [PDB ID: 7SXL] and *Plasmodium falciparum* cysteine protease falcipain‐2 [PDB ID: 1YVB]) were downloaded from pdb data bank ‘https://www.rcsb.org/’,^23^ then they have been made purification using discovery studio 2020 (Figure 2). When purification is done. They have been exported in PDB format for molecular docking. Molecular docking is one of the fundamental tools used in computational drug design.^24^ In our current investigation, we performed blind docking techniques to determine at which site, the ligands formed a bond. Furthermore, the gridbox size was utilized for the receptor ‘Protease Falcipain‐2_(PDB ID: 1YVB), Center *X* = 80.449, Center *Y* = ‐38.2131, Center *Z* = −84.5992, Size *X* = 45.385, Size *Y* = 63.942, and Size *Z* = 44.8354, and for the receptor’ (PDB ID: 7SXL), Center X = 100.9252, Center *Y* = 84.7921, Center *Z* = 1.8829, Size *X* = 61.66958, Size *Y* = 95.8073 and Size *Z* = 61.31644. These grid parameters were generated when we selected maximize in the PyRx application to ensure comprehensive exploration of the potential binding sites and enable analyses of ligand‐receptor interactions. From docking studies, the type of ligand interaction with the target protein, how many non‐bonds are created, and non‐bond distance between ligand and amino acid residues have been analyzed, and the procedure was conducted with the help of PyRx application.^25^, ^26^

Second, it is essential to develop and verify a reliable docking methodology. To accomplish this, when dealing with a target that possesses a ligand co‐crystallized within its binding pocket (78.5, −36.3 and −96.6 for protein *Plasmodium falciparum* cysteine protease falcipain‐2 and 96.1107.2–21.5 for the protein apicoplast DNA polymerase Å via *x*, *y* and *z* coordinates, respectively), it is advisable to extract the co‐crystallized ligand and perform a re‐docking procedure into the same binding pocket. Subsequently, using the initial structure as a reference, align the re‐docked structure and calculate the root mean square deviation (RMSD). A favorable outcome is indicated when the RMSD value registers below 2 Å for a significant portion,^24^ around 10 or more, of the highest‐scoring docked conformers. This achievement validates the quality of the docking procedure.

### 2.3 Molecular dynamics against *Plasmodium falciparum* apicoplast DNA polymerase enzyme (PDB ID: 7SXL)

The structural behaviour of protein‐ligand complexes was evaluated using the GROMACS program (version 2023 with GPU acceleration) on the Linux operating system.^27^, ^28^ Input files for MD simulations in GROMACS were generated using the SwissParam server based on the docking results and built with the CHARMM27 force field.^29^, ^30^ The initial structure of the *Plasmodium falciparum* apicoplast DNA polymerase enzyme (7SXL.PDB) with ligands 03, 05, and standard was solved with SPC waters in periodic and cubic boxes. The solvent systems were then subjected to the steepest descent energy minimization process. The systems were equilibrated in two stages using NPT and NVT ensembles.^31^ All three systems underwent a 2 ns (100,000 steps) equilibration at 300 K and 1 atm in both NPT and NVT ensembles, using a time step of 0.02 fs. This was conducted to ensure that the systems were fully converged prior to the molecular dynamic simulation was run. Finally, production dynamics were performed using GROMACS with a time step of 2 fs for 100 ns. The stability of the protein‐ligand complexes was assessed using the RMSD, root mean square fluctuation (RMSF), and radius of gyration (Rg) calculations. Visual molecular dynamics (VMD) and Pymol software were used for molecular visualization in all simulations.^32^, ^33^

### 2.4 Molecular dynamics simulation against *Plasmodium falciparum* apicoplast DNA polymerase enzyme (PDB ID: 7SXL)

The YASARA dynamics software was implemented to conduct the MD simulation with the help of AMBER 14 force field.^34^ The docked complexes were the earliest cleanup and optimized, as well optimization of the hydrogen bond network was also conducted. GAFF and assigning AM1BCC charges were applied to create the topology files of ligands. During simulation, A cubic simulation cell was developed by the TIP3P solvation model having a periodic boundary condition.^35^ The simulation system was neutralize to provide the physiological conditions, 0.9% NaCl, 310 K and pH 7.4 was maintain.^34^ The particle mesh Ewald (PME) technique was applied to calculate the long‐range electrostatic interaction with an 8.0 cut‐off radius.^36^ The simulation was run with a time step of 2.0 fs. The main form of energy reduction was performed using the steepest gradient methods (5000 cycles) employing simulated annealing techniques. The RMSD, Rg, SASA and hydrogen bond were calculated using the simulated trajectories. The simulation lasted 100 ns, and the simulation paths were captured after every 100 ns.^37^, ^38^, ^39^

### 2.5 Determination of drug likeliness

SwissADME (http://www.swiss‐adme.ch/index.php) is a web‐based internet service that uses the canonical smiles of drug candidates to estimate how likely ligand molecules are to be drugs.^40^ SwissADME let us find out the molecular weight, number of flexible bonds, number of hydrogen bond donors and acceptors, TPSA, consensus Log *p* and absorption score of the ligand. A ligand molecule's draggability can be judged by looking at these things. It helps the medical chemistry process to be able to guess whether or not a medicine will work. The log *p* value is less than 5 if the molecular weight is less than 500 Da,^41^ the number of hydrogen bond donors is less than 5 and the number of hydrogen bond acceptors is less than 10. The ‘rule of five’ can be used to guess how much a molecule will be absorbed or let through when it comes into contact with a lipid membrane in the body.

### 2.6 Determination of ADMET, and pharmacokinetics

ADMET is ‘absorption, distribution, metabolism, excretion and toxicity’. Most failed medication development efforts may be attributed to the drug's harmful effects. Drug research and development relies heavily on ADME/Tox data. Almost half of all unsuccessful medications may be attributed to inaccurate ADME/Tox estimates.^42^ The ADME/Tox qualities are considered while choosing a ligand. ADMET properties may be predicted using the server pkCSM (https://biosig.lab.uq.edu.au/pkCSM/prediction).^43^ A compound's physiochemical qualities and molecular structure are considered when calculating ADMET values.

## 3 RESULTS AND ANALYSIS

### 3.1 Molecular optimizations

**FIGURE 3:**
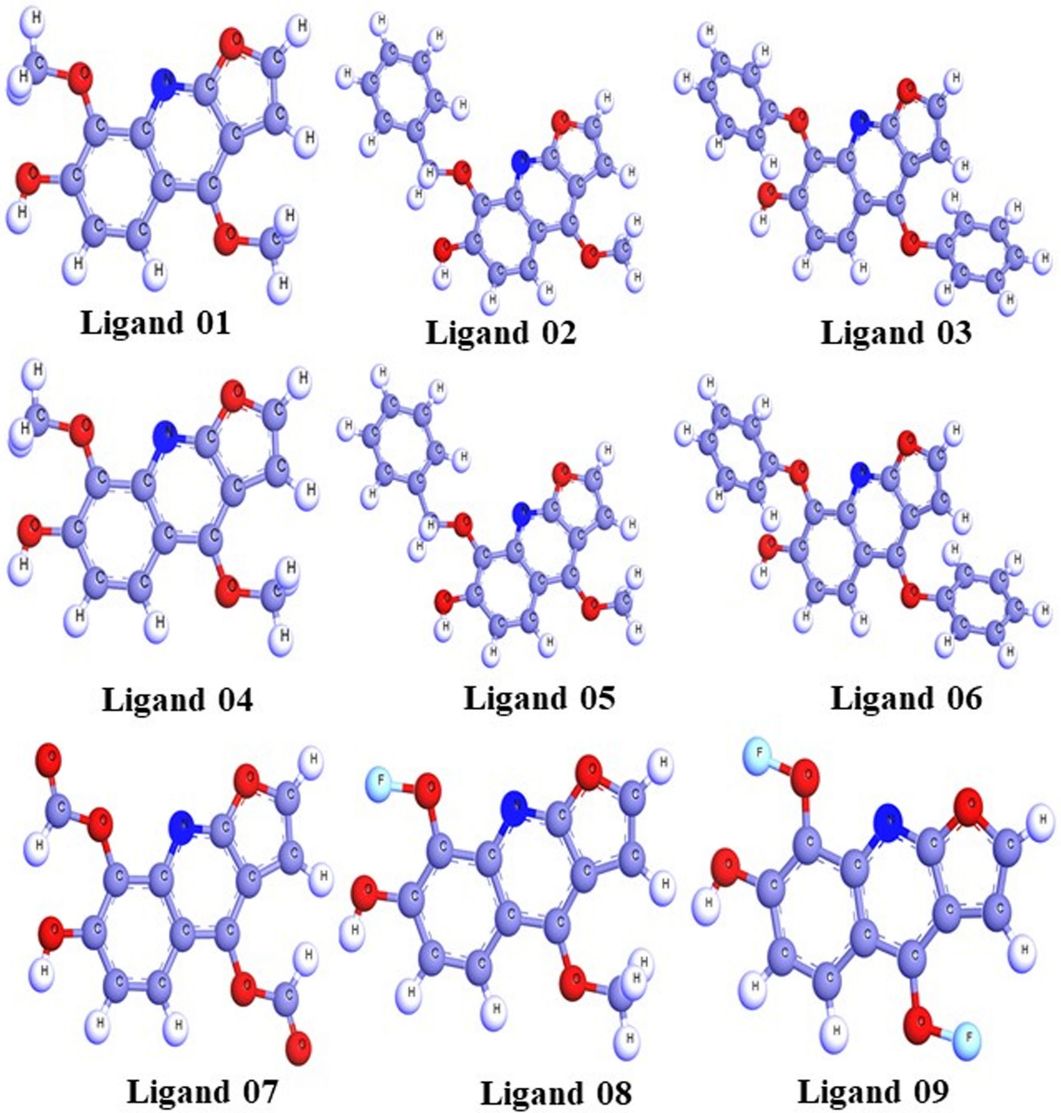
Optimized structure of haplopine and its derivatives.

Developing molecules with desirable properties is one of the most challenging aspects of drug production. A medicine must balance numerous factors, including its physical properties, ADMET properties, safety and effectiveness on its intended target.^44^ The primary objective of molecular optimization is to transform a drug‐like structure into a more functional drug. Before creating a drug, it is necessary to identify the stable combination of molecule structures. Figure 3 depicts the optimal geometries for haplopine derivatives, determined using the DFT method. The enhanced structures of the molecules reveal that their arrangement is highly similar.

### 3.2 Lipinski rule and drug likeness

The Lipinski rule and drug likeness provide valuable information about the earliest phases of drug development and enhance the chances of success. Molecular properties of drugs, such as permeability and bioavailability, depend on factors like molecular weight, the number of hydrogen bond donors and acceptors, the number of rotatable bonds and the partition coefficient (log *p*), as outlined by the Lipinski rule of five. All ligand compounds successfully passed the Lipinski rule without any violations. The ligand compounds have a low molecular weight, which means they are better absorbed, diffused, and transported compared to compounds with higher molecular weights.^45^ Topological polar surface area (TPSA) is a very significant pharmacokinetics property of molecule that informed about polarity of ligand compound. This value is useful to describe the drug transport properties. Polar surface area is the sum of all polar atoms present in molecule mainly oxygen, nitrogen including attached hydrogen.^46^ Bioavailability is an essential factor for any oral drug candidate and high bioavailability score increases binding selectivity profile and decreased undesirable effect of drug. Our all value shows very good biological activities and most of them is around 0.55 or 55%.^47^ These reported molecular properties is represented that the reported molecules might be orally active showing in Table 1.

**TABLE 1:** Data of Lipinski rule.

Ligand no.	Molecular weight	Number of rotatable bonds	Hydrogen bond acceptor	Hydrogen bond donor	Topological polar surface area Å^2^	Consensus Log *p*_o/w_	Lipinski rule		Bioavailability score
							Result	Violation	
Ligand 01	245.23	2	5	1	64.72	2.24	Yes	0	0.55
Ligand 02	321.33	4	5	1	64.72	3.49	Yes	0	0.55
Ligand 03	369.37	4	5	1	64.72	4.66	Yes	0	0.55
Ligand 04	275.21	3	7	2	102.02	1.78	Yes	0	0.56
Ligand 05	305.2	4	9	3	139.32	1.33	Yes	0	0.56
Ligand 06	259.21	3	6	1	81.79	1.92	Yes	0	0.55
Ligand 07	273.2	4	7	1	98.86	1.57	Yes	0	0.55
Ligand 08	249.19	2	6	1	64.72	2.41	Yes	0	0.55
Ligand 09	253.16	2	7	1	64.72	2.55	Yes	0	0.55

### 3.3 Validation of molecular docking using re‐docking

**FIGURE 4:**
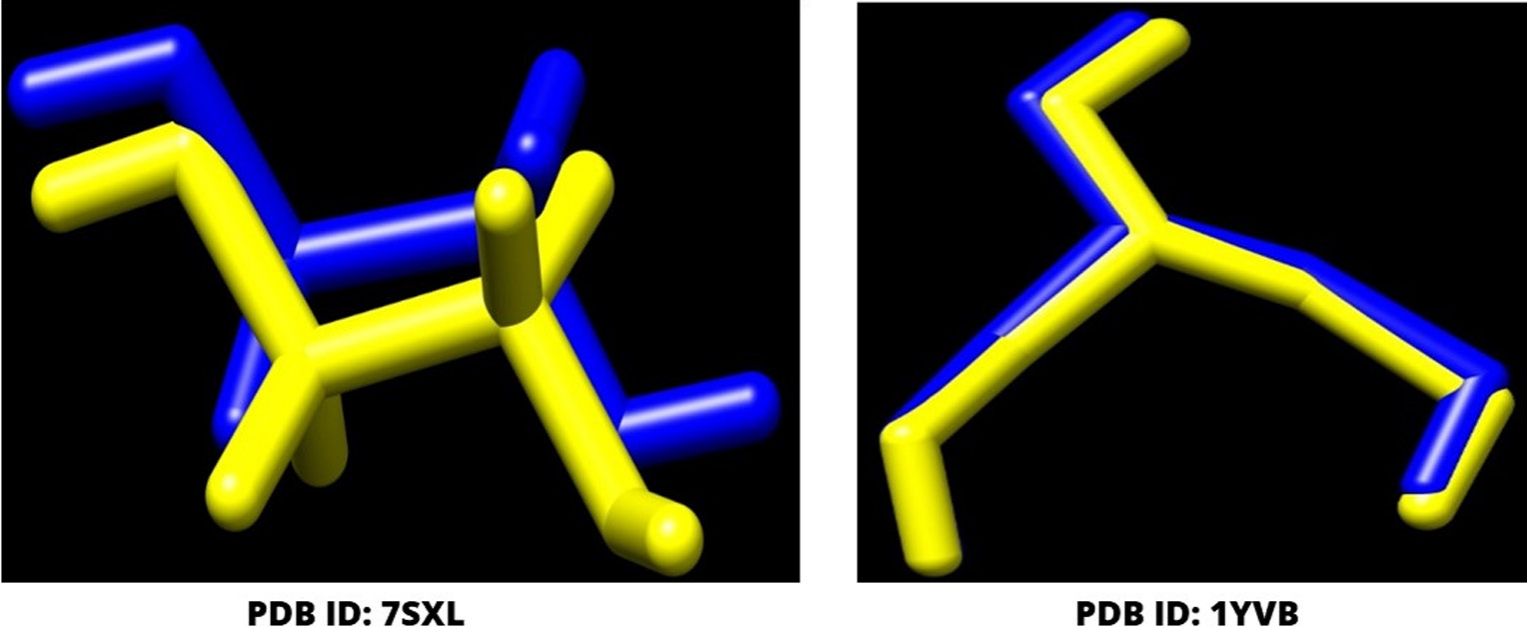
The docking validation was performed by re‐docking the co‐crystal ligand to their corresponding receptor. The original confirmation of each co‐crystal ligand is depicted in blue colour and while the dock pose is illustrated in yellow colour. The RMSD was calculate between the original and dock pose and it was reported as less than 2 Å.

The initial step in a molecular docking analysis involves the careful examination of the structural characteristics of reference ligands (1,2‐ethanediol and glycerol), which are subjected to a process of re‐docking within the active sites of proteins designated as PDB ID: 7SXL and PDB ID: 1YVB, as depicted in Figure 4. This critical stage serves as a foundation for elucidating the ligand‐protein interactions, potential binding modes and stability.

The evaluation of molecular stability is a pivotal consideration in this analysis. Stability, in this context, is inferred from the extent to which the RMSD of the re‐docked molecules deviates from their original positions. A threshold of 2 Å has been established as a criterion for molecular stability. If the RMSD values remain under this threshold, it suggests that the molecules are relatively stable in their re‐docked configurations within the active sites.^48^

In our present investigation, a rigorous exploration of the ligand‐protein interactions has led to the identification of optimal binding poses. These binding poses are characterized by RMSD values of 0.88 Å and 0.15 Å for the PDB ID: 7SXL and PDB ID: 1YVB proteins, respectively. These values denote the degree of convergence between the re‐docked ligand structures and their native conformations within the active sites.

The obtained RMSD values not only provide insights into the stability of the molecular configurations but also serve as a quantitative measure of the accuracy and reliability of the docking process. The achievement of minimal RMSD values underscores the precision of the molecular docking methodology employed and further validates its utility in predicting ligand‐protein interactions by molecular dynamic simulation.

### 3.4 Molecular docking analysis against targeted receptor

Molecular docking is one of the significant tools in computer aided drug design. By this tool binding energy and non‐bond interaction of protein ligand complex can be analysed. Haplopine drug molecules were prepared for performing molecular docking studies against two Malarial target receptors such as Apicoplast DNA polymerase (PDB ID: 7SXL) and *Plasmodium falciparum* cysteine protease falcipain‐2 (PDB ID: 1YVB). Drug molecules were screened based on their binding affinity score. The maximum negative scores molecules that they are the best drug candidate. Normally, −6.00 kcal/mol binding affinity was considered as standard score for an effective drug molecule.^49^ But our drug molecule much better binding affinity, molecule number 03 and 05 show maximum binding energy −8.4 and −8.0 kcal/mol against apicoplast DNA polymerase (PDB ID: 7SXL) target receptor and maximum binding energy against *Plasmodium falciparum* cysteine protease falcipain‐2 (PDB ID: 1YVB) target receptor is −7.8 and −6.7 kcal/mol is seen in molecule 03 and 05. Where control drug molecule shows binding energy −7.7 and −7.2 kcal/mol, respectively which indicate our drug molecule doing better performance against these two targets. So, these designing drug molecule might be a very effective against selected receptor and may promising drug candidate (Table 2).

**TABLE 2:** Binding affinity against targeted protein.

Molecules no.	Apicoplast DNA polymerase (PDB ID: 7SXL)		*Plasmodium falciparum* cysteine protease falcipain‐2 (PDB ID: 1YVB)	
	Binding affinity (kcal/mol)		Binding affinity (kcal/mol)	
	Docking	Re‐docking	Docking	Re‐docking
Ligand 01	−6.8	−6.8	−6.2	−6.2
Ligand 02	−7.6	−7.4	−7.0	−7.1
Ligand 03	−8.4	−8.5	−7.8	−8.0
Ligand 04	−7.6	−7.6	−6.2	−6.3
Ligand 05	−8	−7.9	−6.7	−6.7
Ligand 06	−7.2	−7.0	−6.3	−6.0
Ligand 07	−7.2	−7.0	−6.0	−6.4
Ligand 08	−7.1	−7.1	−6.4	−6.6
Ligand 09	−7.6	−7.6	−6.6	−6.8
Control drug atovaquone	−7.7	−7.9	−7.2	−7.5

Second, we performed docking second times against the mentioned target to determine the precision of the binding. The redocking binding affinity of the mentioned compounds slightly fluctuates and most of the compounds report similar affinity when compared with first docking (Table 3).

**TABLE 3:** H–bonds formed by the ligand molecule with the targeted proteins.

Donor	Acceptor	Occupancy
Ligand‐05		
LIG627‐Side‐O7	THR85‐Main‐O	6.72%
ASN82‐Side‐ND2	LIG627‐Side‐O3	0.20%
LIG627‐Side‐O7	THR85‐Side‐OG1	0.10%
LIG627‐Side‐O3	THR85‐Side‐OG1	2.51%
THR85‐Main‐N	LIG627‐Side‐O3	0.65%
THR85‐Main‐N	LIG627‐Side‐O2	1.15%
THR85‐Side‐OG1	LIG627‐Side‐O2	0.05%
LIG627‐Side‐O6	GLU93‐SideOE2	37.04%
LIG627‐Side‐O6	GLU93‐SideOE1	26.62%
LIG627‐Main‐N	GLU89‐Side‐OE2	26.72%
LIG627‐Main‐N	GLU89‐Side‐OE1	27.82%
Ligand‐stand		
LIG627‐Side‐O2	THR86‐Main‐O	0.30%
ASN196‐Side‐ND2	LIG627‐Main‐O	0.20%
ASN196‐Side‐ND2	LIG627‐Side‐O1	0.10%
LIG627‐Side‐O2	THR85‐Main‐O	2.50%
THR85‐Main‐N	LIG627‐Side‐O2	6.69%

### 3.5 Molecular docking poses and interaction analysis

The lock‐and‐key hypothesis, which was developed by Fischer and was an early explication for the ligand‐receptor binding process, states that the ligand fits into the receptor like a lock and key. This hypothesis served as the foundation for the first docking procedures ever described, and as a result, the ligand and receptor were both regarded as rigid entities in these early docking calculations.^50^ Pymol and BIOVIA Discovery Studio Visualizer and chimeraX software was utilized to visualize the 3D structure of protein‐ligand complex and their non‐bond interaction. From docking result, the docked complex with maximum binding affinity was taken into further molecular dynamic simulation analysis to validate the result. In Figure 3, (a) Docking pocket (b) 2D picture of ligand and protein interaction is represented. The docking pocket is seen how closely bind ligands with targeted proteins, and 2d images shows the active amino acid residues, which form during the binding interaction.

**FIGURE 5:**
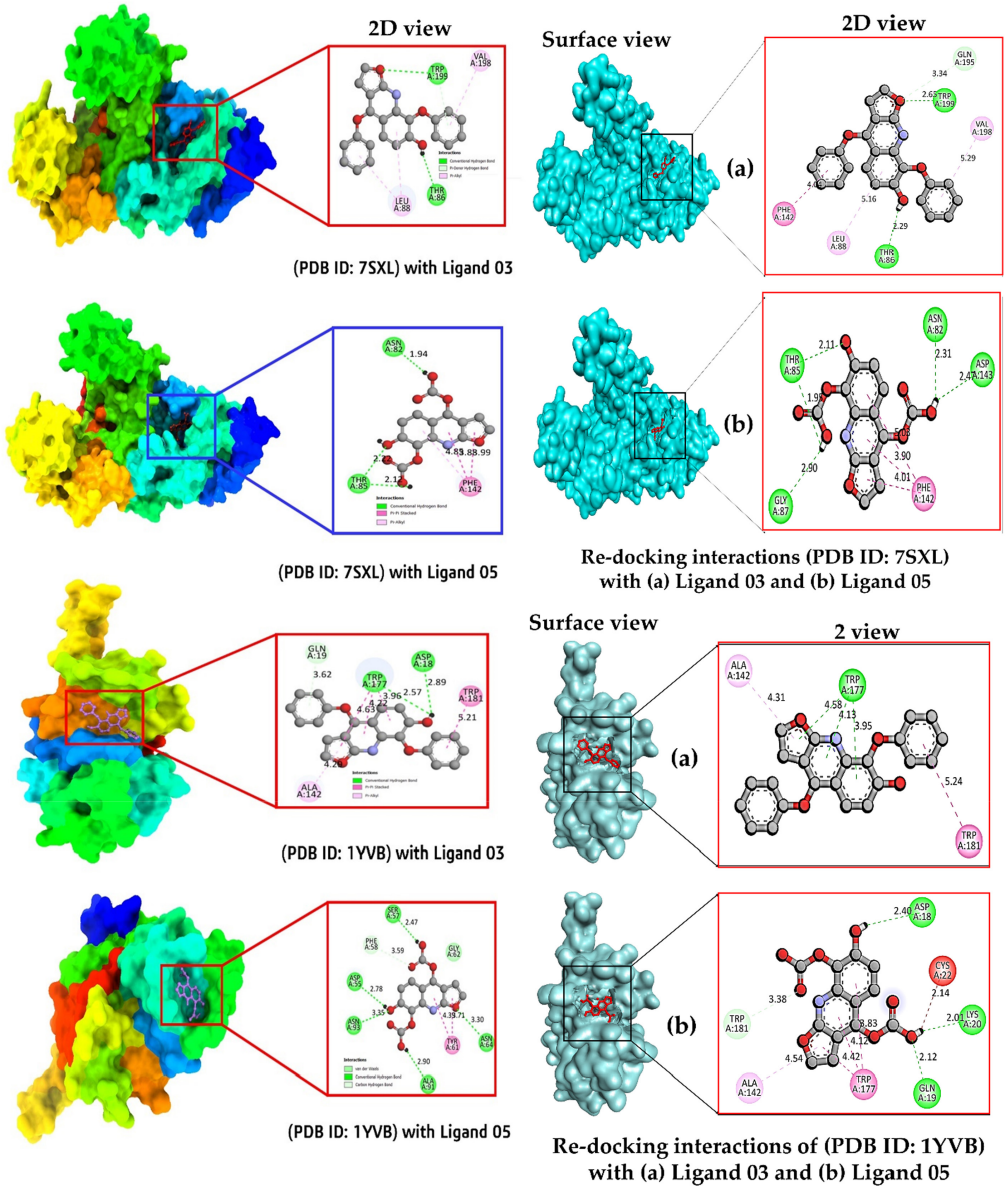
Docking interactions between the proposed compound with target receptor (left site), and re‐docking interactions (right site).

Second, we conducted redocking again to compare the active site of the initial docking (left site) and re‐docking interactions (right site). After that, it is seen that the active site between the initial docking and re‐docking interactions has slightly changed. For the Ligand 03 complex with PDB: 7SXL, the documented residues are TRP A:199, VAL A:198, THR A:86, LEU A:88 and ASN A:82 in the initial docking, while the active re‐docking site includes THR A:86, LEU A:88, TRP A:199, VAL A:198 and PHE A:142 for the Ligand 03 complex with PDB: 7SXL. Similarly, for the Ligand 05 complex with PDB: 7SXL, the initial docking has residues THR A:85, ASN A:82 and PHE A:42, whereas the active re‐docking site includes THR A:85, ASN A:82, ASP A:143, PHE A:142 and GLY A:87. In comparison with them, ASP A:143 and PHE A:142 are present in re‐docking but missing in initial docking. Similarly, slightly different active sites are observed for the complex of initial docking and re‐docking of *Plasmodium falciparum* cysteine protease falcipain‐2 (PDB ID: 1YVB) Figure 5.

### 3.6 Molecular dynamics simulation analysis against apicoplast DNA polymerase (PDB ID: 7SXL).

Molecular dynamics simulations (MDS) aid in determining the conformational strength of receptors and drug candidates by modelling the system at an atomistic scale. To examine the stability of a ligand with a targeted protein macromolecule, MD simulation is an excellent and unique approach.^51^ In this case, a 100 ns MD simulation was performed to analyse the complex structure of the selected compounds. This was done to assess how effectively the drug candidates could bind to the protein and the active site cavity. The results of the MD simulation are described based on the RMSD, RMSF and Rg.

#### 3.6.1 Root means square deviation analysis

**FIGURE 6:**
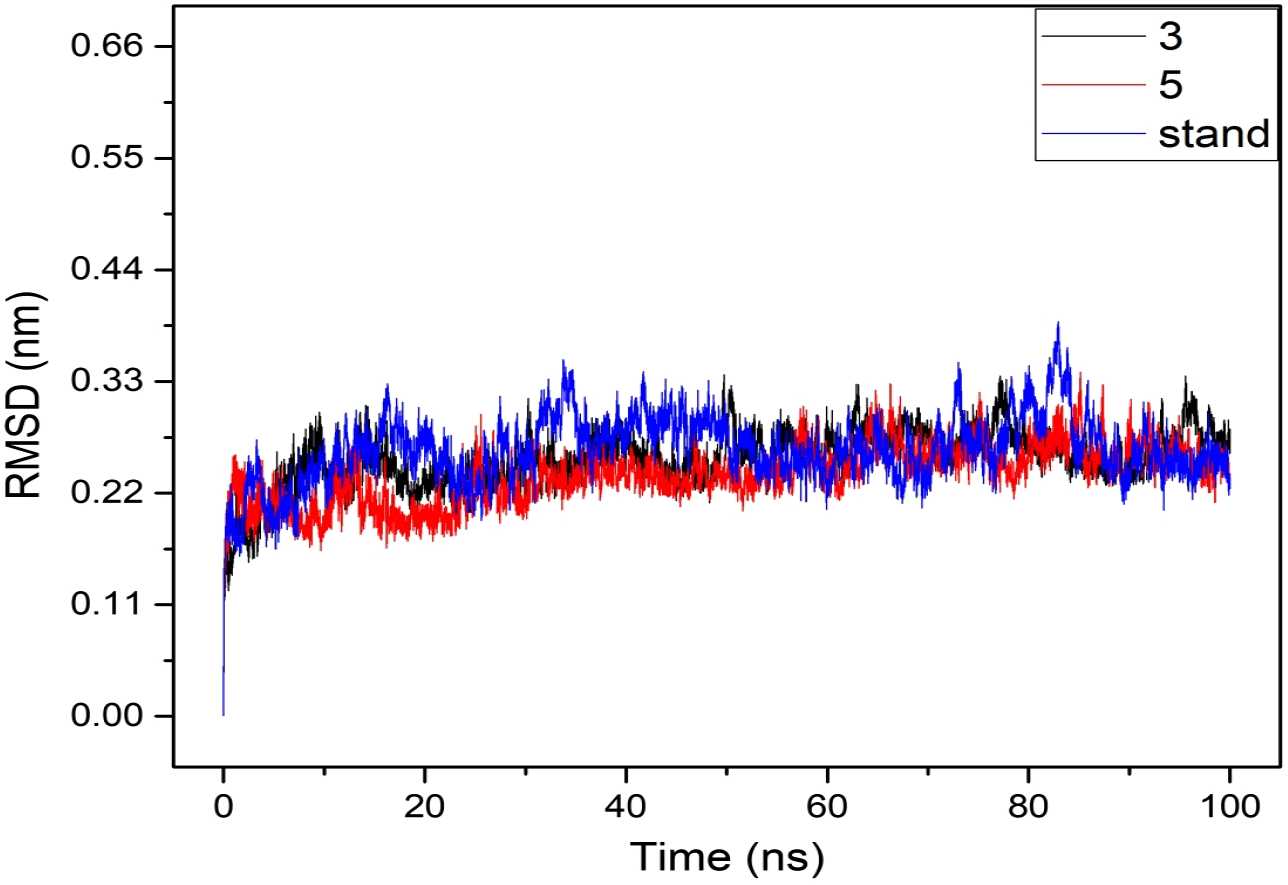
The graphs show the RMSD values for the three compounds in complex with the targeted receptor apicoplast DNA polymerase enzyme (PDB ID: 7SXL) in 100 ns MDS assessments, where the selected two drug candidate compounds Ligand 03, Ligand 05 and the control drug in associated with the protein are exhibited by black, red and blue colour, respectively.

The RMSD of a protein–ligand complex system enables the determination of the average distance caused by the displacement of a chosen atom over a specified time period. It is essentially the square root of the mean of squared errors that is utilized to quantify the difference between two values (observed value and estimated value). The mean or average value changes from one frame to another within the range of 1–5 Å or 0.1–0.5 nm is considered acceptable, while a value outside this acceptable range indicates a significant conformational shift in the protein. The RMSD of the drug candidate compounds—Ligand 03 (black colour), Ligand 05 (red colour) and the control drug (blue colour)—in their complex structure has been compared with the selected receptor, Apicoplast DNA polymerase (PDB ID: 7SXL), to observe changes in their arrangement, as depicted in Figure 6. The RMSD for the three compounds was in the range of 0.1–0.3 nm with slight fluctuations, whereas the ligand compounds exhibited fewer fluctuations compared to the control drug when interacting with the targeted protein.

#### 3.6.2 Root mean square fluctuation

**FIGURE 7:**
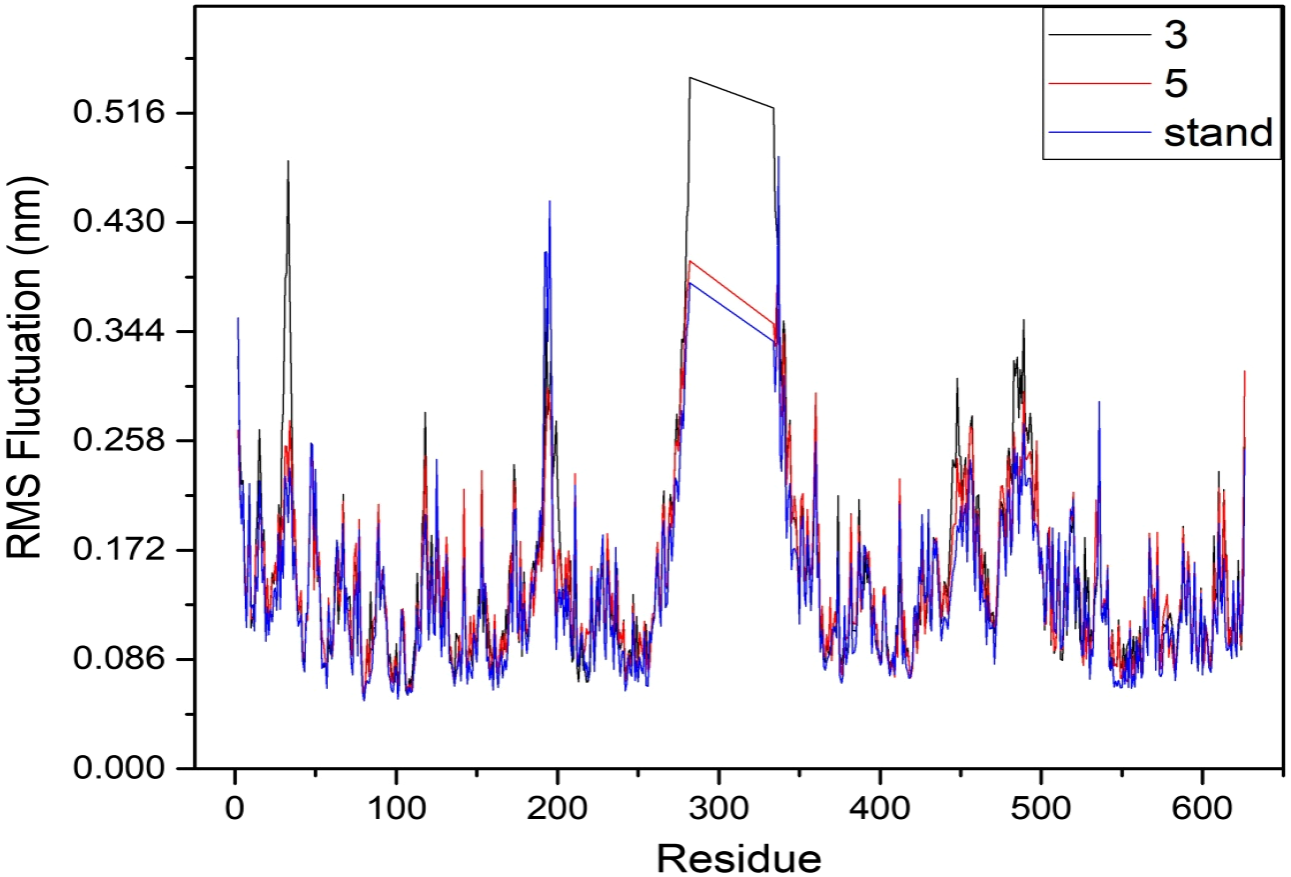
The graphs display RMSD values for three compounds complexed with the target receptor apicoplast DNA polymerase enzyme (PDB ID: 7SXL) during 100 ns MDS assessments. The selected drug candidate compounds, Ligand 03 and Ligand 05, along with the control drug associated with the protein, are represented by black, red and blue colours, respectively.

The RMSF is crucial for observing local protein changes, as it allows the calculation of the average change observed across a large number of atoms. This assessment helps determine the displacement of a given atom in comparison to the reference structure. RMSF is a numerical calculation akin to the RMSD, both of which are important for characterizing proteins. They aid in understanding the flexibility and fluctuation of protein residues during simulations. Consequently, RMSF values were computed for the drug candidate compounds: Ligand 03 (black), Ligand 05 (red) and the control drug (blue) bound to the Apicoplast DNA polymerase enzyme (PDB ID: 7SXL). This analysis aimed to investigate the impact of attaching the selected ligand compounds to specific residue positions on the protein's structural flexibility. This process is illustrated in Figure 7.

It was revealed that the most rigid secondary structural components, such as alpha‐helices and beta‐strands, exhibit minimal variations across the entire amino acid residue range of the target protein. Given that the protein possesses both N‐ and C‐terminal domains, most of the protein's variations may be located at these terminal points. Consequently, it can be inferred that the displacement of a single atom experiences low fluctuation probability in the simulated environment for the investigated ligand compounds, as illustrated in Figure 7.

#### 3.6.3 The radius of gyration analysis

**FIGURE 8:**
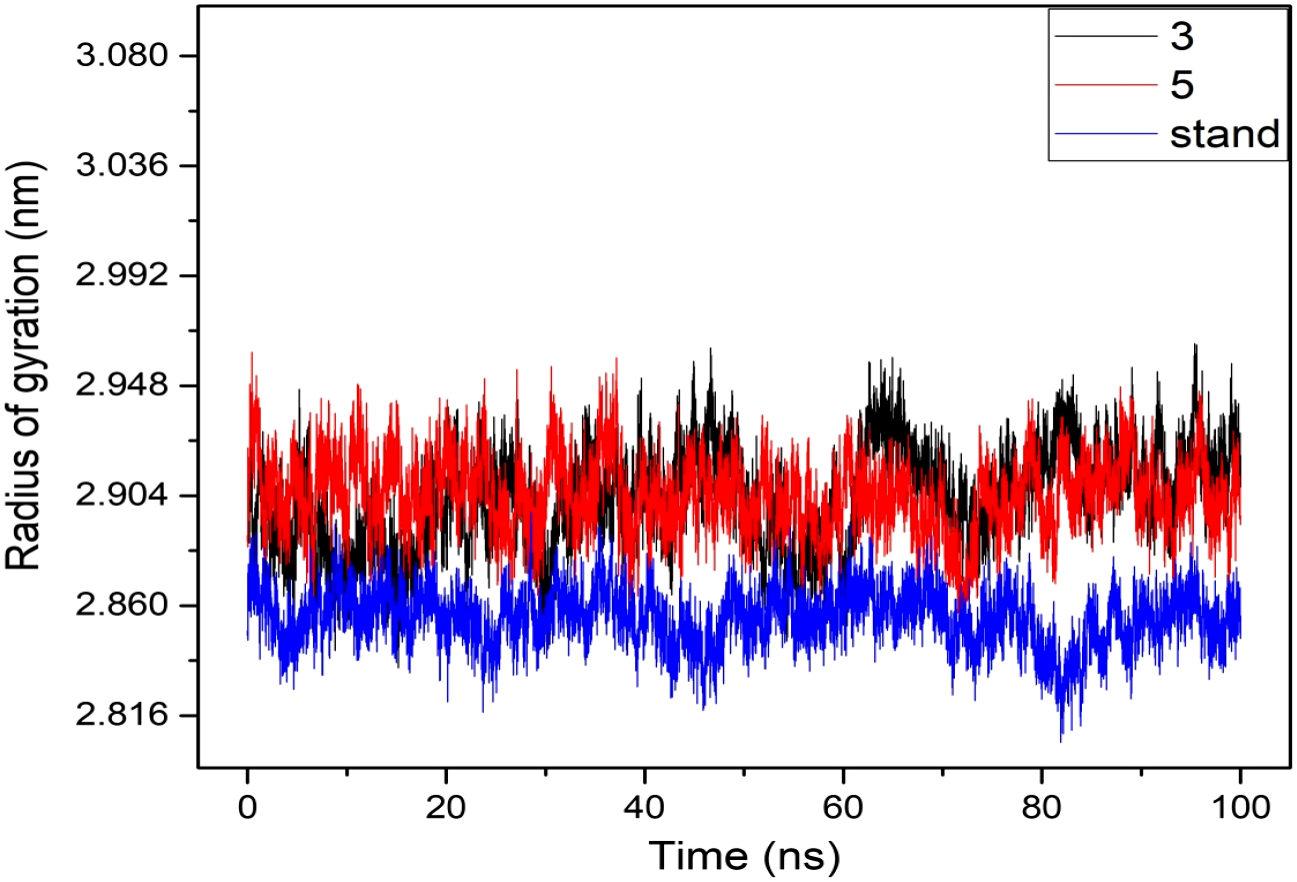
The graphs show the radius of gyration (Rg) values for the three compounds in complex with the targeted receptor Apicoplast DNA Polymerase Enzyme (PDB ID: 7SXL) in 100 ns MDS assessments, where the selected two drug candidate compounds Ligand 03, Ligand 05 and the control drug in associated with the protein are exhibited by black, red and blue colour, respectively.

The Rg for a protein‐ligand interaction complex can be defined as the arrangement of its atoms around its axis. Calculating Rg is one of the most crucial indicators to consider when assessing the structural behaviour of a macromolecule, as it reflects changes in the compactness of the complex throughout the simulation. Consequently, as depicted in Figure 8, the stability of the drug candidate compounds—Ligand 03 (black), Ligand 05 (red) and the control drug (blue)—in interaction with the target protein was investigated in terms of Rg over a 100 ns simulation period. The average Rg values for the drug candidate compound Ligand 03 (black), Ligand 05 (red) and the control drug (blue) in association with the apicoplast DNA polymerase enzyme (PDB ID: 7SXL) ranged from 2.816 to 2.960 nm. This range indicates that the protein's binding site did not undergo significant structural changes upon ligand binding.

### 3.7 Principal component analysis

**FIGURE 9:**
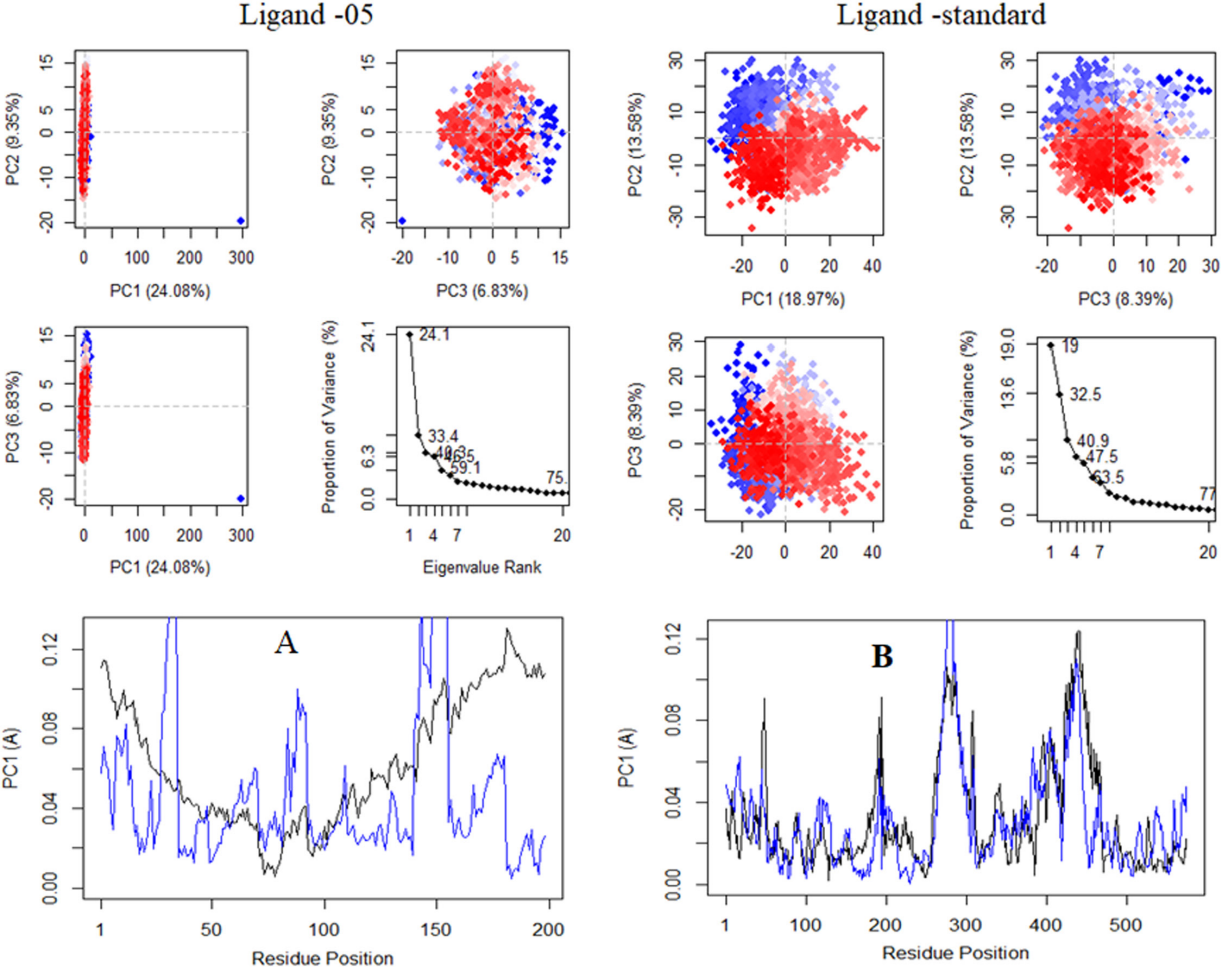
Graphically represented of principal component analysis (PCA).

PCA was utilized to examine the domain dynamics within the receptor‐ligand complex during a 100‐ns simulation (Figure 9). The outcomes were presented in terms of eigen fractions, which indicate the proportion of variance, derived from a covariance matrix comprising 20 eigen models. The atomic backbone of the complex system underwent PCA calculations using three conformations—PC1, PC2 and PC3—via normal mode MD.

The results of the PCA revealed conformational changes across all clusters. Particularly, the blue region exhibited the most significant movements, the white region showed intermediate movements and the red region demonstrated the least flexible movements.

Figure 9 displays the results of principal component analysis (PCA) and distance covariance matrix (DCCM) analysis conducted on the MD simulation trajectories of the 7SXL@05 and 7SXL@stand complexes. The conformational transformation of the Ligand‐05 and Ligand‐stand systems was explored by obtaining PCA scatter plots through the projection of simulated trajectories into the two‐dimensional subspace defined by the first three eigenvectors (PC1, PC2 and PC3). The progression of time is represented by the continuous colour spectrum, which goes from blue to white to red. The starting timescale is represented by the colour blue, the intermediate timescale by the colour white and the ultimate timescale by the colour red. The RMSF of residue contribution to PCA is depicted in Figure 9 (left and right site), where the black and blue lines represent PC1 and PC2, respectively. DCCM plots for Ligand‐05 and Ligand‐stand. The positive numerical quantity denotes the presence of positively correlated movements in the system, as indicated by the cyan coloration. Conversely, the negative numerical quantity signifies the existence of anti‐correlated motions, which are represented by the pink hue.

The analysis presented in Figure 9 demonstrates that the top 20 principal components (PCs) of the Ligand‐05 system and the Ligand‐stand system contributed to 75% and 77% of the overall variance, respectively. This suggests that the Ligand‐05 system had a more restricted phase space and less performance flexibility compared to the Ligand‐stand system.

In comparison to the PCA plots of Ligand‐stand and Ligand‐05, the PC1 cluster exhibited the greatest variability, accounting for 24.08% and 18.97% of the variance, respectively. The PC2 cluster demonstrated 9.35% and 13.58% variability, while the PC3 cluster exhibited minimal variability, accounting for only 6.83% of the variance for Ligand‐05. The low degree of variability exhibited by PC3 for Ligand‐05, when compared to the PC1 and PC2 clusters, suggests that the binding of Ligand‐05 is highly stable, and the structure is compact. Additionally, RMSF analysis conducted on the PCA indicated that the flexibility of PC1 and PC2 was reduced in comparison to the Ligand‐stand.

### 3.8 Dynamic cross‐correlation matrix

**FIGURE 10:**
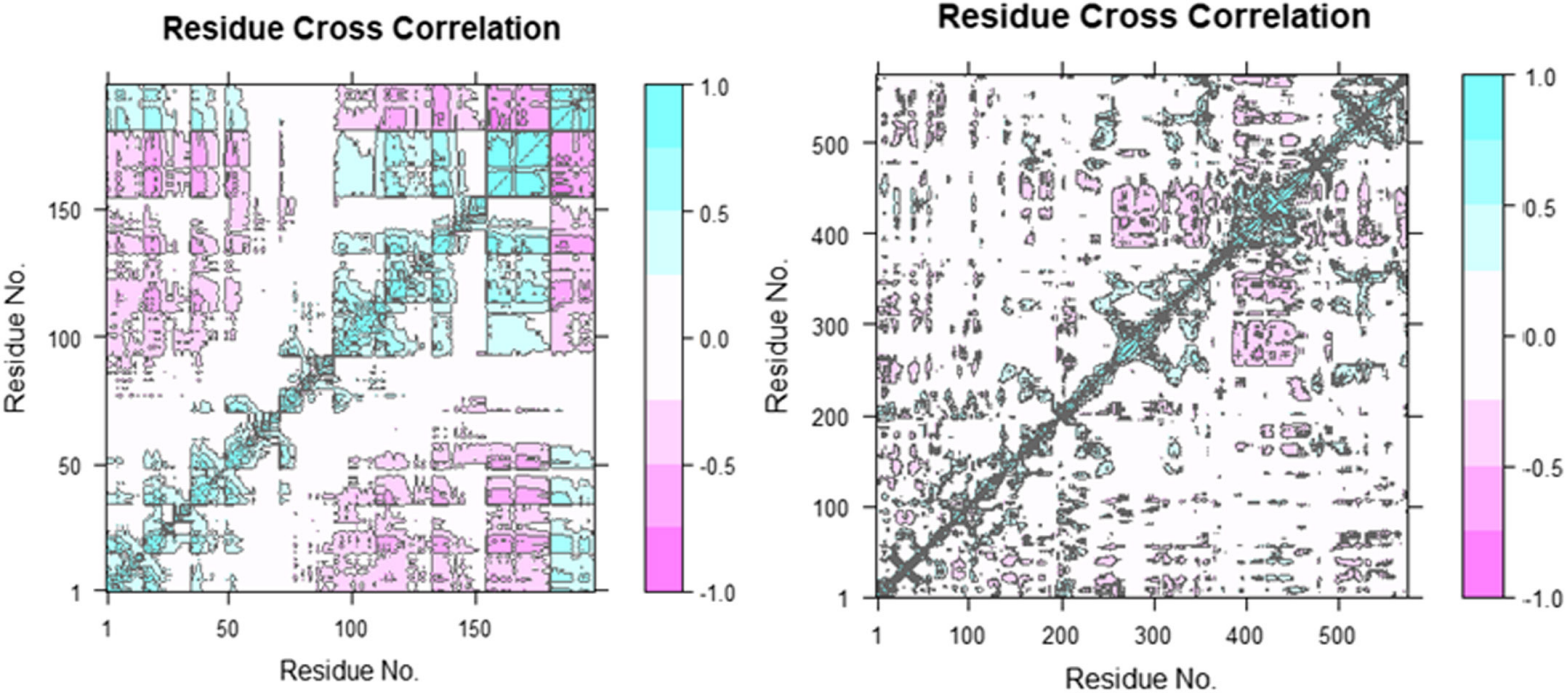
Graphically illustrate of dynamic cross‐correlation matrix (DCCM).

To investigate the effect of ligands on the conformational dynamics of the 7SXL protein, DCCM analyses were conducted on all C atoms in the 7SXL‐05 and 7SXL‐stand complex systems by extracting the last 10 ns of simulated trajectories. The two‐dimensional diagrams of the dynamic cross‐correlation matrix (DCCM) depicted the interrelated movements among amino acid residues throughout the entirety of the simulation procedure (Figure 10). The DCCM demonstrated a comprehensive correlation, encompassing a range of values from −1.0 to 1.0, with the former represented by a dark purple hue and the latter by a dark blue hue. It was determined that different shades of colour correspond to varying degrees of connection between residues, and that the darker the colour, the greater the strength of the link. The correlation coefficient, ranging from −1 to 1, indicated that a positive correlation denoted that the residues moved in the same direction, whereas a negative correlation indicated that the residues moved in the opposite direction. Upon examining the DCCM diagrams of the two systems, it was observed that the correlated movements of the two systems exhibited notable dissimilarities. Compared with the ligand‐05 system, both the positive correlation motions of the entire ligand‐stand system and the negative correlation motions had changed significantly. The DCCM analysis of Ligand‐stand indicates a significant reduction in correlated motions upon the binding of the ligand. The DCCM analysis of Ligand‐stand indicates a significant reduction in correlated motions upon the binding of the ligand.

### 3.9 Hydrogen bond analysis

**FIGURE 11:**
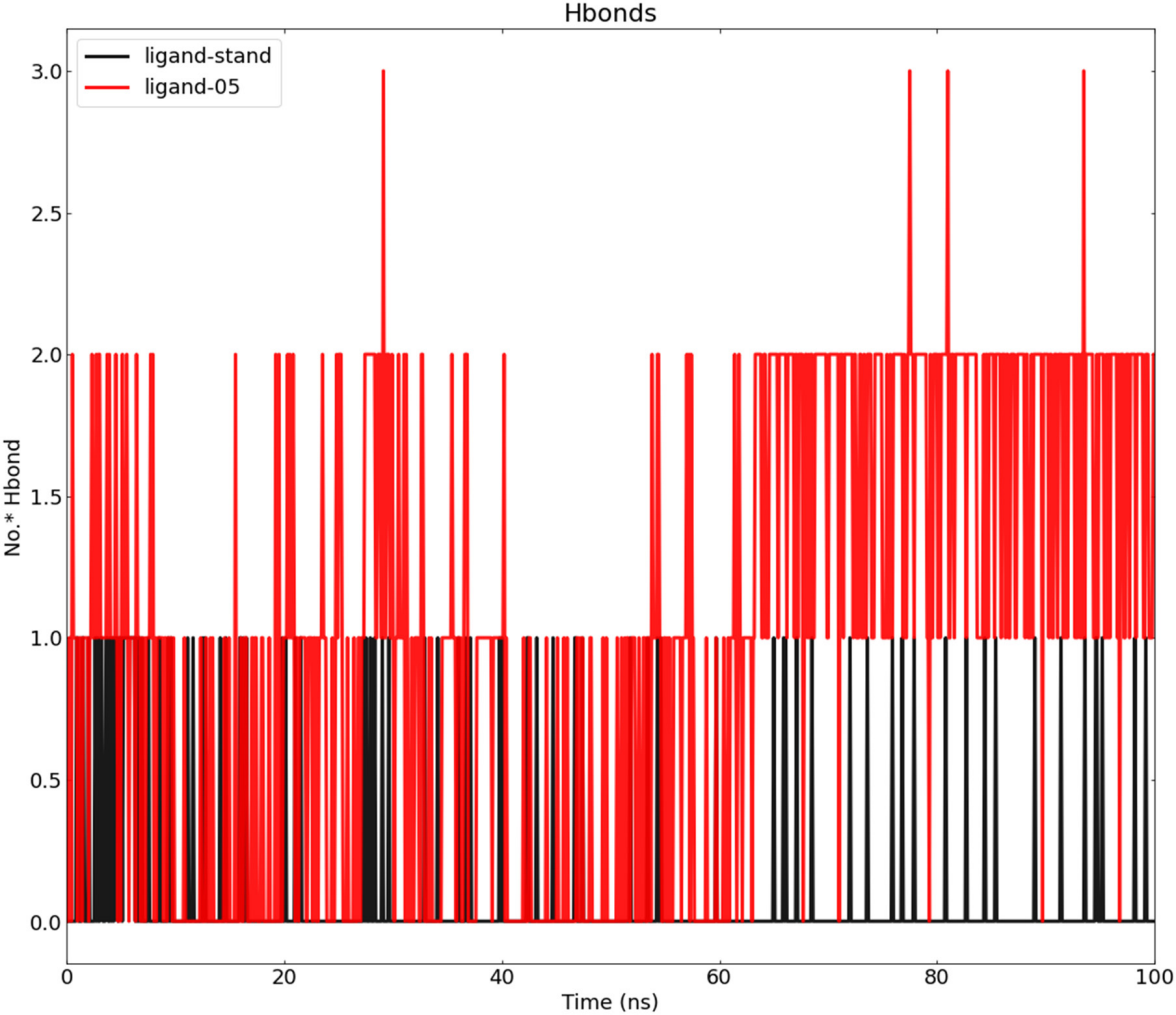
SASA analysis for the complex of Ligand‐stand (black) and complex with ligand‐05 (red).

Calculations and a graph of the H‐bonds that the ligand molecules (Ligand‐05 and Ligand‐stand) formed with the proteins are depicted in Figure 11. Intermolecular H‐bonds between the protein and ligand are essential for the stability of protein‐ligand complexes. Throughout the whole 100 ns simulation period, the degree of stability of the H‐bond network that was created between proteins (Ligand‐05 and Ligand‐stand) and ligands was analysed and calculated. The anticipated outcome was that the quantity of produced H‐bonds would correlate with the duration of the simulation, thereby establishing the stability of the system during the simulation.

In this study, the criteria for identifying hydrogen bonds were established as having an acceptor‐donor distance of less than 0.30 nm and an angle greater than 120 degrees. It is worth noting that a distance of 0.30 nm is a frequently utilized threshold for hydrogen bond distance in academic literature. The frames utilized for this computation were obtained at intervals of 2 ps from the complete 100‐ns MD trajectory. Because of their strength, hydrogen bonds are deserving of study because they contribute to bonding. Utilizing the VMD H‐bonding analysis tool, all potential H‐bonding interactions between the two specified regions, in this case, the protein and the ligand, have been investigated over time. The output values are used to calculate the sum of H‐bonds and the occupancy rate. The H‐bond analysis tool provides these values in the ‘Percentage occupancy of the Hbond’ output. The H‐bond analysis tool gives these figures in the ‘Percentage occupancy of the H bond’ output.

Figure 11 shows the number of hydrogen bonds (Ligand‐05 and Ligand‐Stand) that contributed to the stability of the complexes during the course of the 100 ns.

During the entire duration of the MD simulation, it was observed that the quantity of H‐bonds in the ligand‐bound states exhibited a constant fluctuation, indicating the preservation of H‐bonds in docking structures. It was also found that other H‐bonds existed. The graphical representation illustrates that the ligand‐05 complex exhibits a greater number of hydrogen bonds over the course of the simulation, in contrast to the ligand‐stand complex which displays a lower count of hydrogen bonds. The standard ligand compound formed only three H‐bonds in the clamped simulation, and none of these were seen in the simulation result. The simulation results indicate that H‐bonds were less prevalent in standard‐ligand systems, with the majority of them emerging at 10 ns, 20–40 ns, and predominantly occurring within the final 30 ns. However, a greater number of hydrogen bonds were observed in the ligand‐05 complex. The majority of the hydrogen bonds exhibit particularly strong intensity during the initial 60 ns of the simulation. During the docking simulation, we could only see seven of these hydrogen bonds in action. Interactions with GLU 93, GLU 89 and THR 85 residues served to preserve the stability of the protein‐ligand‐05 complex during MD in the instance of the ligand‐05 complex. Because ligand‐05 forms the hydrogen bond with GLU 93, it possesses hydrogen bonds with an occupancy level that is 37.04% higher than average. Then there is an occupancy of 27.82% with GLU 89, where ligand‐05 acts as an acceptor, and there is occupancy of 26.72% with GLU 93. The ligand‐05 complex exhibited a higher number of hydrogen bonds, totaling 30 more than the other complex. Additionally, the compound demonstrated the formation of hydrogen bonds during the docked simulation in comparison to the standard ligand. The standard ligand possesses a total of just five hydrogen bonds, with the occupancy level being at its maximum (6.69%) in the region where hydrogen bonds are formed between the ligand and THR 85. Then, occupancy of 2.50%, where standard‐ligand acts as an acceptor of THR 85; occupancy of 0.30% with THR 86; occupancy of 0.20% with ASN 196; and occupancy of 0.10% with ASN 196 (Table 3). During the docked simulation, not a single one of the five hydrogen bonds was able to establish. It is concluded that the ligand‐05 complex exhibits greater stability than the standard‐ligand complex during the process of MD.

### 3.10 Solvent accessible surface area analysis

**FIGURE 12:**
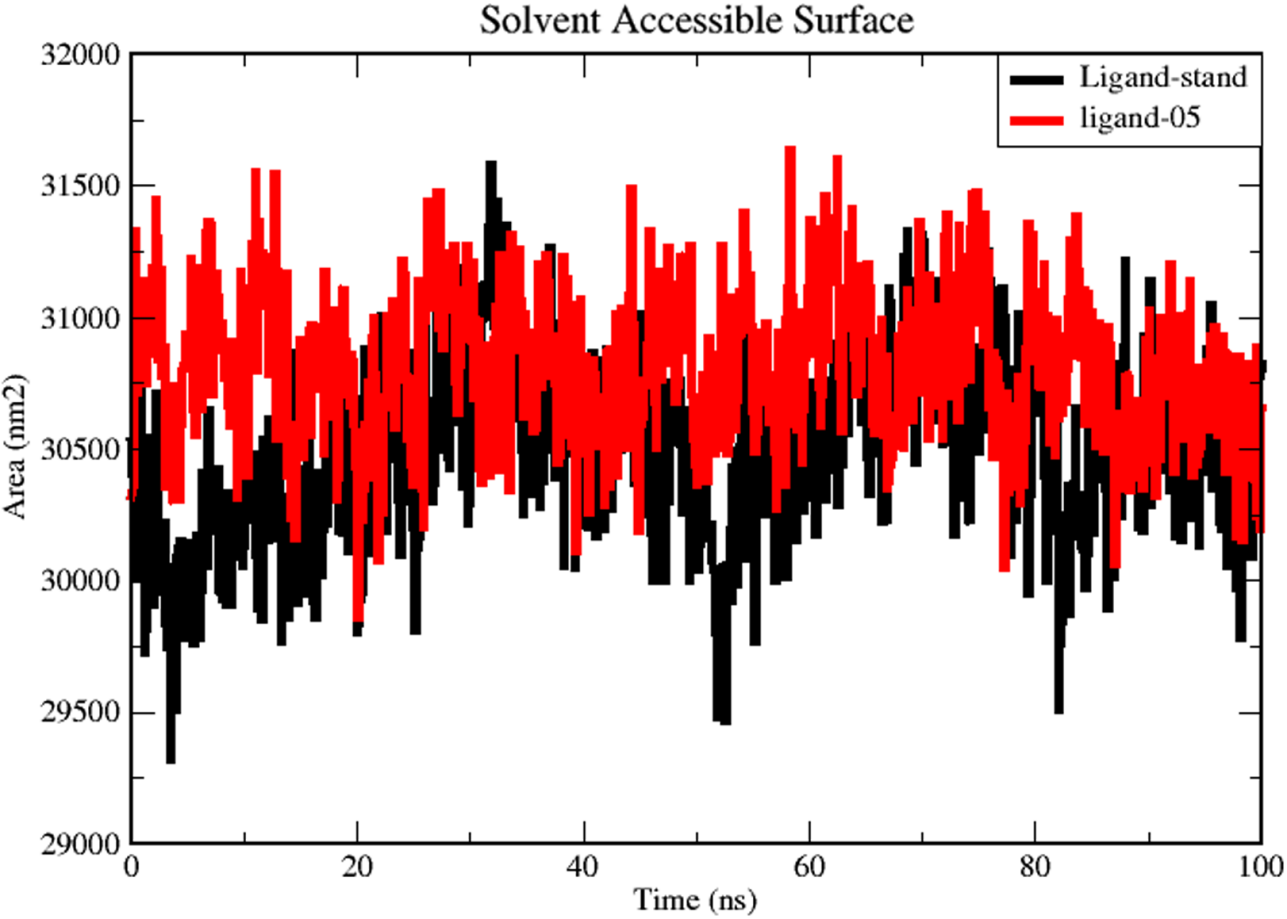
SASA analysis for the complex of Ligand‐stand (black) and complex with ligand‐05 (red).

When examining whether the ligand is retained within the shallow binding pocket or whether it is released from the binding cavity, the information collected by SASA will be helpful. The SASA for ligand‐05 and stand‐ligand complexes was estimated to be 30,650 based on Figure 12. The average SASA for ligand‐05 was calculated to be 31,200, while the average SASA for stand‐ligand was calculated to be 30,650. It has been shown that the stand‐ligand complex displays a lower SASA in comparison to the ligand‐05 complexes; this indicates that the stand‐ligand complex is responsible for inducing conformational alterations.

### 3.11 MD simulation of the complexes between cysteine protease falcipain‐2 (PDB ID: 1YVB) and two best docked ligands

The RMSD, Rg, solvent accessible surface area (SASA), hydrogen bonds and RMSF of the cysteine protease falcipain‐2 and the top two docked ligands (L02 and L03) complexes were analysed in comparison to the standard atovaquone.

**FIGURE 13:**
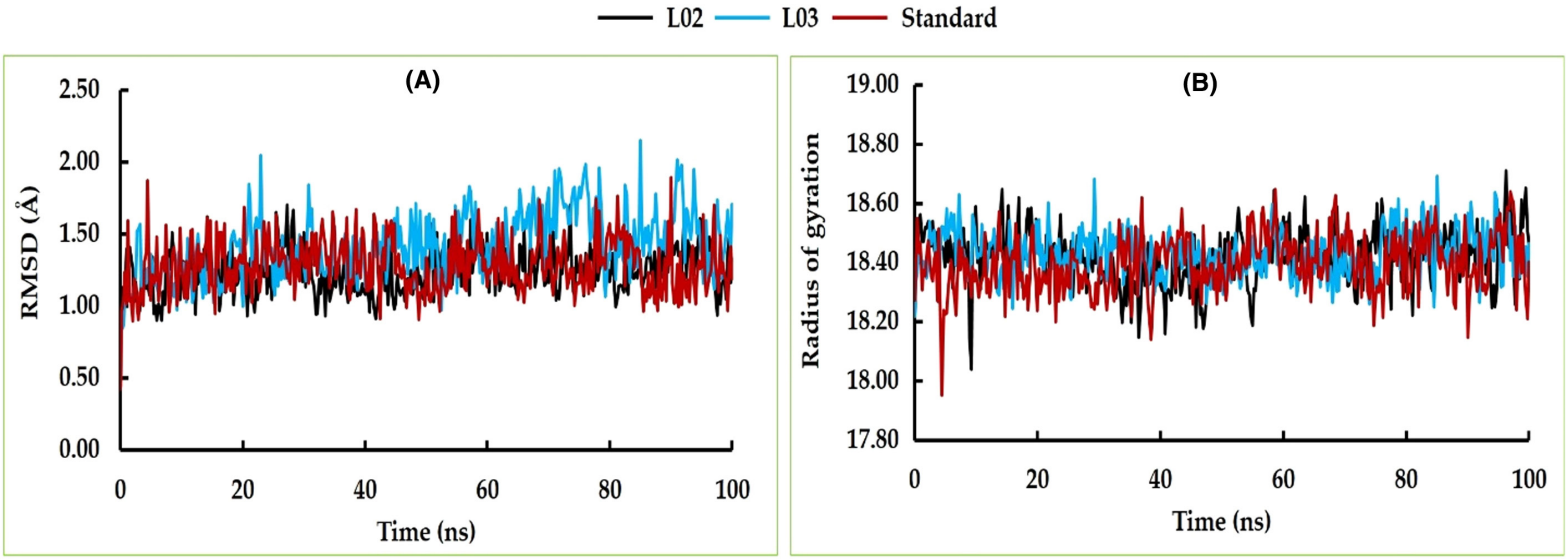
The RMSD (A) and radius of gyration (B) of cysteine protease falcipain‐2 (PDB ID: 1YVB) with L02 and L03 ligands compared to the standard.

The average RMSD values for L02 and L03 were 1.239 and 1.419 Å, respectively, which are nearly similar to the standard atovaquone with 1.277 Å. This suggests structural similarity and stability comparable to the standard. Both ligands, L02 and L03, exhibited strong RMSD profiles throughout the 100 ns simulation period. Although L03 showed slight fluctuations between 55 and 90 ns with an upward trend, it did not significantly affect their stability. Notably, these two ligands displayed nearly identical RMSD stability profiles when compared to the standard complex. This indicates that the structures of L02 and L03 exhibited substantial stability and maintained consistent structures throughout the entire simulation duration (Figure 13A).

The analysis of Rg in MD simulations provides valuable insights into characterizing and quantifying the structural changes, folding dynamics, stability and interactions of molecules. In this study, we observed a similar Rg profile and stability level for both complexes throughout the entire simulation period, similar to the standard (Figure 13B). The consistent Rg values indicate a robust level of inherent structural stability in both complexes.

**FIGURE 14:**
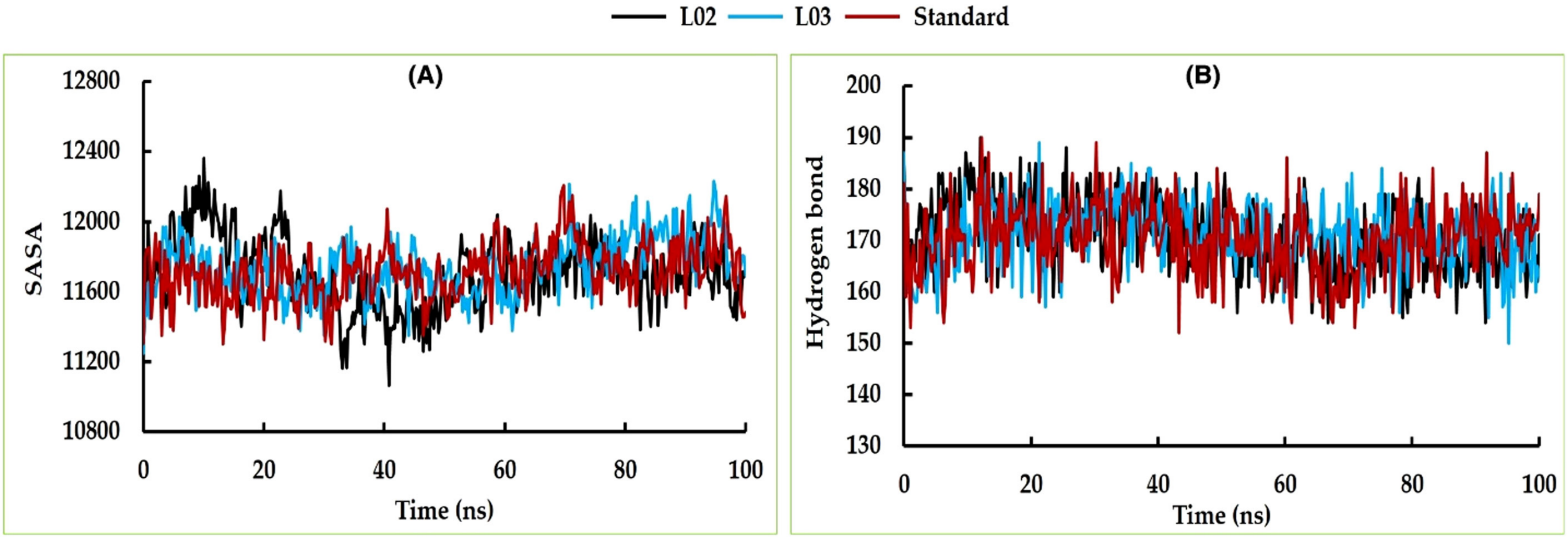
The MD simulation analysis of SASA (A) and hydrogen bond (B) of complexes between cysteine protease falcipain‐2 (PDB ID: 1YVB) and L02, L03 ligands compared to the standard.

Calculating the SASA provides valuable insights into the surface exposure, binding site dynamics, stability, interactions and behaviour of the complex in a solvent environment. Based on our comprehensive analysis, it is evident that the SASA profiles of L03 exhibited a remarkable degree of similarity with standard. Conversely, for L02, a distinct pattern is observed: an initial decline is noted up to approximately 35 ns, followed by a subsequent shift in the opposite direction up to 45 ns, culminating in a convergence with the stability exhibited by the remaining two complexes (Figure 14A).

From the beginning of the simulation period all complexes displayed slight increasing trend up to 10 ns and subsequently maintained the stable hydrogen bond profile throughout 100 ns simulation period (Figure 14B). This finding suggests that a consistent hydrogen bond pattern emerged across the complexes, signifying a stabilized configuration and sustained molecular interactions throughout the extended simulation timeframe.

**FIGURE 15:**
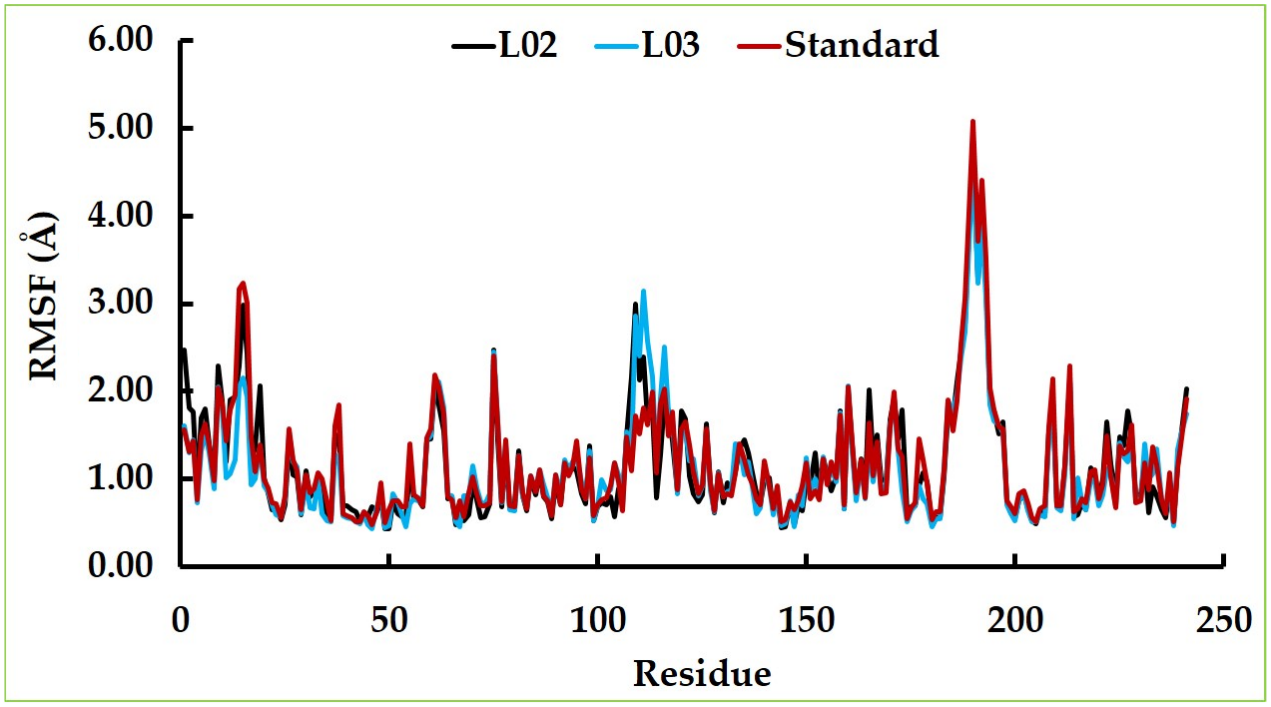
The RMSF analysis of top two docking complexes compared to the standard.

Through our investigations, the analysis of RMSF unveiled a consistent trend across the residues of all three examined complexes. Notably, these residues exhibited a state of structural stability that persisted throughout the entirety of the 100 ns simulation duration. However, it is noteworthy that a nuanced degree of fluctuation was observed within the residues encompassing glu16, glu17, asn18, ser109, asp110, pro190, leu191, thr192, lys193 and lys194 (Figure 15).

### 3.12 Frontier molecular orbitals and chemical reactivity descriptor

The calculation of chemical reactivity descriptors including εHOMO, εLUMO, ∆E gap, chemical potential (μ), electronegativity (x), hardness (η) and softness (σ) of the drug molecule compounds is presented in Table 5. For enhanced chemical stability, it is crucial to have a lower energy gap between HOMO and LUMO. A wider energy gap signifies a higher atomic system and chemical instability.^52^, ^53^ The energy gap of the frontier molecular orbitals (FMOs) HOMO and LUMO is used to assess atomic electrical transport characteristics, and a smaller energy gap facilitates the development of drug interactions with proteins. In Table 4, the HOMO and LUMO energy gaps of the mentioned compounds range from 2.70 to 4.15 eV. Compound 09 possesses the minimum energy gap with the maximum softness and minimum hardness. In contrast, compound 04 has a higher energy gap between HOMO and LUMO, along with the minimum softness and maximum hardness. Notably, a relatively lower softness value for a compound implies a quicker rate of degradation due to faster disintegration compared to other compounds. The FMO analysis aids in determining the chemical reactivity and active sites of the compound, which can interact with proteins to exhibit protein‐drug interactions. The values of HOMO‐LUMO energy gap, hardness and softness presented in Table 5 collectively suggest that the compound has the potential to inhibit the target disease.

**TABLE 4:** Chemical reactivity descriptor data.

S/N	I = −HOMO	A = −LUMO	E(gap) = I−A	Hardness	Softness
Ligand 01	−5.09	−1.17	3.92	1.96	0.5102
Ligand 02	−5.44	−1.40	4.04	2.02	0.4950
Ligand 03	−5.68	−1.880	3.8	1.9	0.5263
Ligand 04	−5.73	−1.574	4.15	2.078	0.4812
Ligand 05	−6.07	−2.07	4.0	2.0	0.5000
Ligand 06	−5.73	−1.63	4.1	2.05	0.4878
Ligand 07	−6.32	−2.34	3.98	1.99	0.5025
Ligand 09	−6.84	−4.14	2.70	1.35	0.7407

**TABLE 5:** Summary of ADMET profile.

S/N	Absorption			Distribution		Metabolism		Excretion		Toxicity			
Ligand no	Water solubility Log S	Caco‐2 permeability ×10^−6^	Human intestinal absorption (%)	VDss (human)	BBB permeability	CYP450 1A2 inhibitor	CYP450 2D6 substrate	Total clearance (mL/min/kg)	Renal OCT2 substrate	AMES toxicity	Max. tolerated the dose log mg/kg/day	Skin sensitization	Hepatotoxicity
01	−2.935	1.165	93.89	0.081	Yes	Yes	No	0.615	No	Yes	0.46	No	No
02	−4.162	1.053	94.52	−0.23	No	Yes	No	0.553	No	No	0.466	No	Yes
03	−4.085	0.992	94.12	−0.192	Yes	Yes	No	0.611	No	No	0.622	No	No
04	−2.91	0.138	66.07	−1.041	No	No	No	0.493	No	No	1.479	No	Yes
05	−2.919	−0.879	47.06	−1.219	No	No	No	0.388	No	No	1.223	No	Yes
06	−2.76	1.201	94.29	−0.056	No	Yes	No	0.599	No	No	0.55	No	Yes
07	−2.787	0.635	88.91	−0.721	No	Yes	No	0.676	No	No	0.72	No	Yes
08	−3.065	1.158	92.32	0.012	Yes	Yes	No	0.503	No	Yes	0.49	No	No
09	−3.679	1.222	93.36	−0.159	Yes	Yes	No	0.448	No	Yes	0.265	No	No

### 3.13 Frontier molecular orbitals (HOMO and LUMO)

**FIGURE 16:**
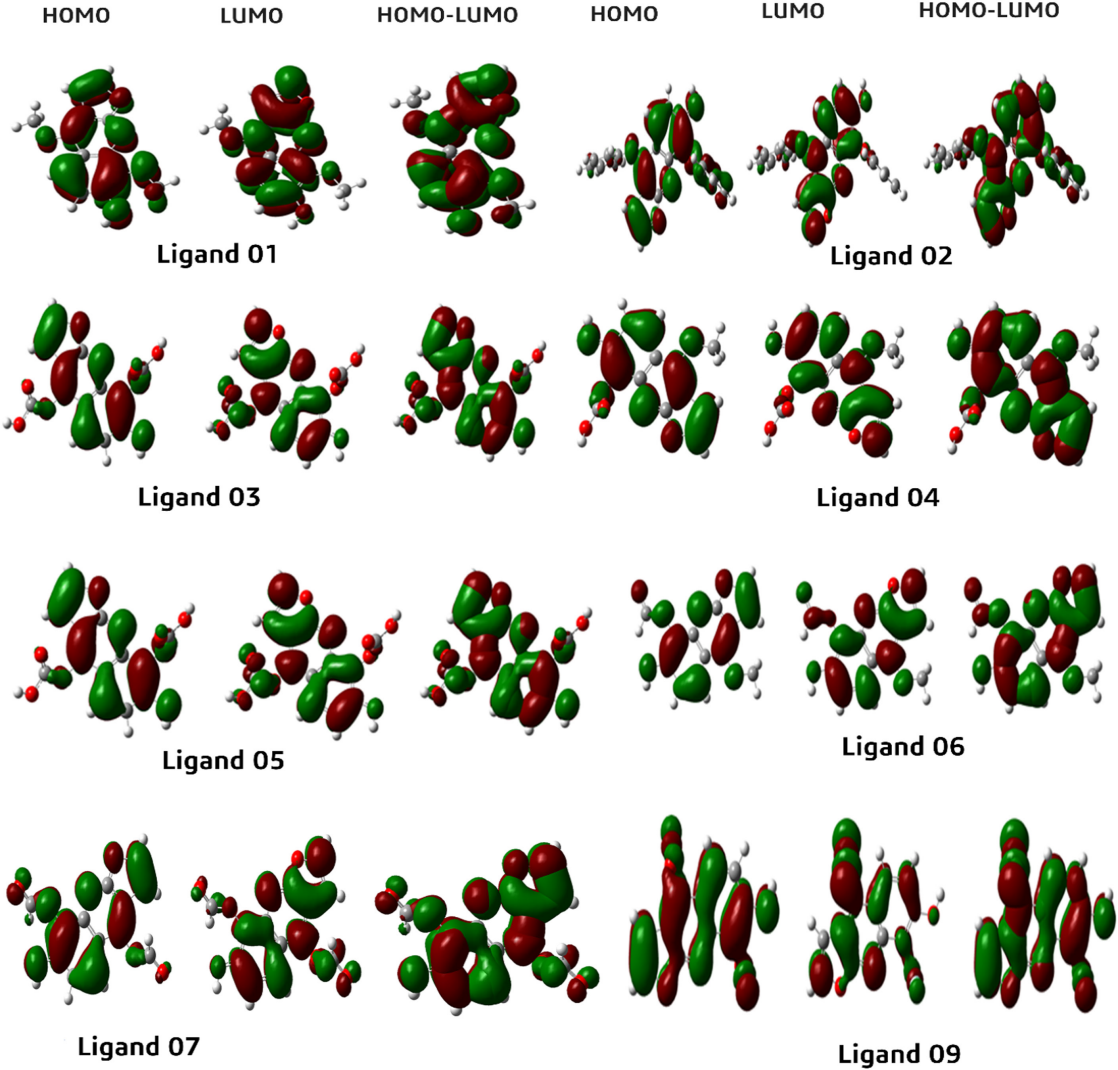
HOMO and LUMO graphical illustration.

The FMOs were utilized to evaluate the kinetics and identify specific regions where proteins might fold, becoming active pharmacophores with active functional groups. The orbital geometry of our compound was determined using DFT. HOMO indicates an orbital with electron‐dense regions, while LUMO indicates an electron‐deficient region. HOMO stands for the highest occupied molecular orbital, while LUMO stands for the lowest unoccupied molecular orbital.^54^ In Figure 16, the blue colour indicates the positive terminal of the orbital in LUMO, while the pink colour represents the negative node. In our Figure 16, the LUMO is located in the benzene ring where no functional groups or atoms are present. Conversely, the LUMO is situated in a position where the benzene ring contains functional groups like oxygen, hydroxide and nitrogen.

### 3.14 ADMET data investigation

ADMET data investigation has a significant impact on pharmacology, toxicology and pharmacokinetics, particularly in drug compound selection. This computational tool enables us to choose drug compounds with favourable pharmacological properties such as intestinal absorption, blood–brain barrier (BBB) permeability, toxicological potential and metabolic stability, among others. ADMET data plays a pivotal role in the virtual screening procedure.

Water solubility, indicated by Log S, reflects the molecules' solubility in water at 25°C. Water‐soluble drugs generally have higher absorption rates than lipid‐soluble ones. All our ligands exhibit efficient water solubility. For orally administered drugs, Caco‐2 permeability plays a crucial role. The Caco‐2 cell line consists of human epithelial colorectal adenocarcinoma cells and is used to assess permeability. All ligand compounds show very high Caco‐2 permeability except ligand 05. Human intestinal absorption is vital for orally administered drugs, predicting the proportion of a compound absorbed through the human small intestine. If this value is less than 30%, it is considered to be poorly absorbed. In Table 5, all ligands show high absorption values, ranging from a maximum of 94.525% to a minimum of 47.064%.

The volume of distribution (VDss) is a theoretical volume indicating the uniform distribution of a drug at a specific concentration in human blood plasma. A higher VDss implies greater distribution in tissues than in plasma. If the Log VDss value is < −0.15, it is considered low, and if >0.45, it is considered high. In Table 5, our VDss values range from 0.081 to −1.219, with the maximum and minimum values, respectively. The ability of a drug to cross the BBB enhances its effectiveness, efficiency, and reduces toxicities and side effects. Ligand 05 has the lowest BBB permeability, while ligand 03 has the highest.

Drug metabolism studies are based on the cytochrome P450 enzyme substrate and inhibitor, mainly found in the liver. These enzymes can deactivate or activate certain drugs. Thus, cytochrome P450 inhibitors and substrate enzymes play a crucial role in drug metabolism. Our drug compounds did not interact with the CYP450 2D6 substrate, while all ligands interacted with CYP450 1A2 inhibitors except ligands 04 and 05. The excretion of drug compounds from the body occurs through various pathways, primarily the liver and kidneys. Total clearance rate estimates the drug's elimination rate per unit time, considering both hepatic and renal elimination. Table 5 presents the total clearance rates for our compounds, ranging from a maximum of 0.676 to a minimum of 0.388. The organic cation transporter 2 (OCT2) is another critical parameter influencing renal clearance.

AMES toxicology testing is vital in determining the mutagenic potential of drug compounds. All our ligands exhibit negative results for mutagenic potential, except ligands 01, 08 and 09. The maximum tolerated dose provides an estimate of the toxic dose threshold for human exposure to chemicals. This information is crucial for Phase 1 clinical trials to determine the maximum recommended dose. All ligand compounds show negative results for skin sensitization. Hepatotoxicity, or liver injury, is a major safety concern during drug development. Table 5 indicates that ligands 01, 03, 08 and 09 exhibit negative results for hepatotoxicity.

### 3.15 Molecular electrostatic potential analysis

**FIGURE 17:**
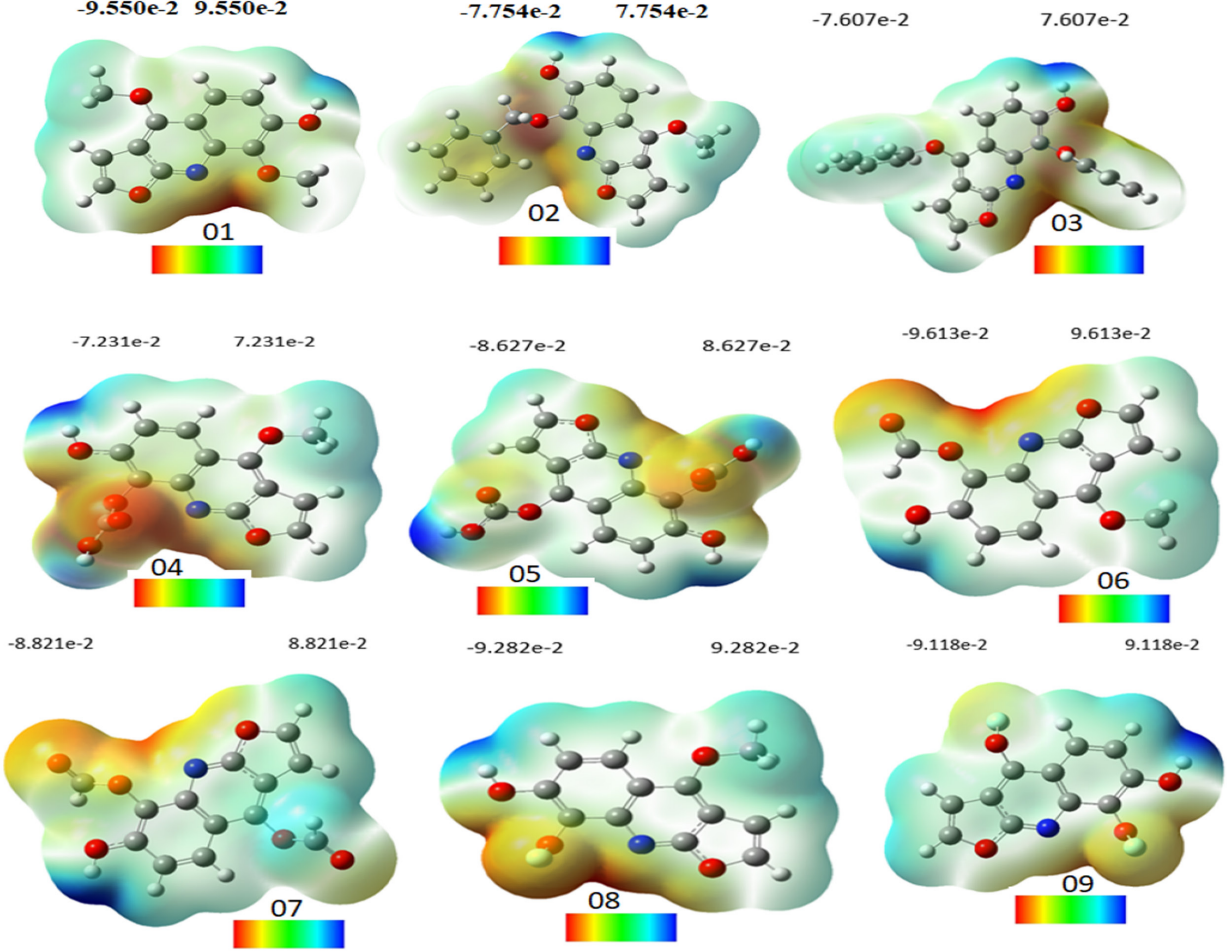
Molecular electrostatic potential diagram.

Molecular electrostatic potential (MEP) provides comprehensive insights into investigations pertaining to chemical reactivity or the biological activity of a substance. The spatial distribution and values of the electrostatic potential primarily determine the primary event of a chemical reaction, dictating the attack of electrophilic or nucleophilic agents.^55^ The binding of a substrate at the active site of a receptor is primarily attributed to the three‐dimensional distribution of the electrostatic potential. The MEP maps for the compounds under study have been generated using the B3LYB/6–311 (d, p) basis sets, as depicted in Figure 17. The mentioned figure illustrates that the MEP's negative regions, denoted by the colour red, are associated with electrophilic reactivity, while the positive regions, indicated by the colour blue, are linked to nucleophilic reactivity.

Compound (1) exhibited a range of units spanning from −9.550 to 9.550 units, with colours arranged in the order of red, yellow, green and blue. The range of units for compound (2) was observed to be between −7.754 and 7.754, while for compound (3), the range was found to be between −7.607 and 7.607. Similarly, for compound (4), the range of units was noted to be between −7.231 and 7.231, and for compound (5), the range was found to be between −8.627 and 8.627. Compound (6) exhibited a range of units between −9.613 and 9.613, while for compound (7), the range was observed to be between −8.821 and 8.821. Compound (8) showed a range of units between −9.282 and 9.282 and for compound (9), the range was found to be between −9.118 and 9.118.

In Compound 1, positive charges are primarily localized on the hydrogen of the hydroxyl group (H_2_), while electron‐negative atoms of oxygen and nitrogen display a concentration of negative charges, with the most negative sites (indicated in red‐orange) situated therein. In Compound 2, the hydroxyl group exhibits a preponderance of positive charges on the hydrogen atoms, whereas the electron‐negative oxygen atom bears the majority of negative charges, as indicated by the red‐orange regions. For compound 3, the positive charges are primarily located on the hydrogen of the hydroxyl group (H_2_), while the negative charges are concentrated on the electron‐negative atom oxygen located in the aromatic ring, where the most negative sites (shown in red‐orange) are located. For compound 4, positive charges are mostly located on the hydrogen (H_2_) of the hydroxyl group, as in the other three compounds, while negative charges are concentrated on the electron‐negative atom oxygen located in the carbonyl group, where the most negative sites (shown in red‐orange) are located.

Based on the MEP maps depicted in Figure 17, it can be observed that the positive region of Compound 5 is primarily concentrated on atoms H28, H29, H27 and H22, which exhibit higher colour intensity owing to the presence of carbonyl groups. Consequently, there are four nucleophilic attack positions on compound 5. The MEP map of molecule II indicates that the regions of negative charge are predominantly localized on atoms O21, O22 and O16. Based on the MEP maps presented in Figure 17, it can be observed that the negative potential region of compound 6 is predominantly concentrated on the carbonyl groups, specifically atoms O19 and O16, as well as the nitrogen atom present in the aromatic ring. Conversely, positive charges are primarily localized on the hydrogen atom of the hydroxyl group (H_2_).

Compound seven exhibits nucleophilic and electrophilic properties identical to those of compound six. For compound 8, positive charges are mostly located on the hydrogen (H_2_) of the hydroxyl group, while negative charges are concentrated on the electron‐negative atom fluorine located in the carbonyl group, where the most negative sites (shown in red‐orange) are located. In compound 9, akin to compound 8, the hydroxyl group's hydrogen (H_2_) bears the majority of positive charges, whereas the carbonyl group's electron‐negative atom fluorine harbours the negative charges, with the most negative sites (depicted in red‐orange).

## 4 CONCLUSION

This advanced computational approach explored the potential of haplopine derivatives as targeted inhibitors against Apicoplast DNA polymerase and *Plasmodium falciparum* cysteine protease falcipain‐2, which are vital targets in the quest for new antimalarial drugs. The results, derived from molecular docking and MD simulations, illustrated that ligand 03 and 05 displayed robust binding affinities to the target proteins, surpassing the binding of the control drug atovaquone. Throughout the 100 ns simulation period, the ligand‐protein complexes remained stable and retained their structural integrity. This stability was affirmed by analyses involving RMSD, RMSF, Rg and PCA, which detected conformational changes in the ligand‐protein interactions, indicative of their dynamic behaviour. Furthermore, the haplopine derivatives, including ligands 03 and 05, adhered to the Lipinski rule and exhibited favourable drug‐like attributes. ADMET predictions suggested that these compounds hold promise as safe and effective antimalarial drugs, displaying no signs of toxicity. Hydrogen bond analysis provided additional support for the stability of the ligand‐protein interactions. In summary, these findings underscore the potential of haplopine derivatives as promising candidates for the development of targeted antimalarial drugs. Through their specific inhibition of Apicoplast DNA polymerase and *Plasmodium falciparum* cysteine protease, these compounds have the potential to disrupt the life cycle and metabolic pathways of *Plasmodium* spp., offering a novel strategy to combat drug‐resistant malaria. However, further experimental investigations are imperative to validate the efficacy, bioavailability, and pharmacokinetic properties of these derivatives. The computational insights furnished by this study contribute significantly to the ongoing initiatives aimed at discovering innovative and effective malaria therapies. The ultimate goal is to eradicate this life‐threatening infectious disease and enhance global public health.

## AUTHOR CONTRIBUTIONS

**Shopnil Akash:** Conceptualization (equal); data curation (equal); investigation (equal); methodology (equal); software (equal); validation (equal); visualization (equal); writing – original draft (equal). **Guendouzi Abdelkrim:** Conceptualization (equal); formal analysis (equal); investigation (equal); methodology (equal); resources (equal); writing – original draft (equal). **Imren Bayil:** Data curation (equal); formal analysis (equal); methodology (equal); resources (equal); writing – original draft (equal). **Md. Eram Hosen:** Data curation (equal); formal analysis (equal); methodology (equal); validation (equal); visualization (equal); writing – original draft (equal). **Nobendu Mukerjee:** Formal analysis (equal); investigation (equal); methodology (equal); software (equal); visualization (equal); writing – original draft (equal). **Abdullah F. Shater:** Investigation (equal); resources (equal); software (equal); supervision (equal); validation (equal); visualization (equal); writing – review and editing (equal). **Fayez M. Saleh:** Formal analysis (equal); methodology (equal); resources (equal); supervision (equal); validation (equal); writing – review and editing (equal). **Ghadeer M. Albadrani:** Data curation (equal); investigation (equal); software (equal); supervision (equal); validation (equal); visualization (equal). **Muath Q. Al‐Ghadi:** Software (equal); supervision (equal); validation (equal); writing – review and editing (equal). **Mohamed M. Abdel‐Daim:** Supervision (equal); validation (equal); writing – review and editing (equal). **Tuğba Taşkin Tok:** Supervision (equal); validation (equal); writing – review and editing (equal).

## FUNDING INFORMATION

No specific funding received for this study.

## CONFLICT OF INTEREST STATEMENT

The authors declare that there is no conflict of interest.

## Data Availability

All data are given in manuscript
